# Gene expression profiling reveals different pathways related to Abl and other genes that cooperate with c-*Myc *in a model of plasma cell neoplasia

**DOI:** 10.1186/1471-2164-8-302

**Published:** 2007-08-31

**Authors:** Eun Sung Park, John D Shaughnessy, Shalu Gupta, Hongyang Wang, Ju-Seog Lee, Hyun Goo Woo, Fenghuang Zhan, James D Owens, Michael Potter, Siegfried Janz, J Frederic Mushinski

**Affiliations:** 1 Laboratory of Cancer Biology and Genetics, Center for Cancer Research, National Cancer Institute, NIH, Bethesda, MD 20892 USA; 2 Donna and Donald Lambert Laboratory of Myeloma Genetics, Myeloma Institute for Research and Therapy, University of Arkansas for Medical Sciences, AR 72205 USA; 3 Laboratory of Experimental Carcinogenesis, Center for Cancer Research, National Cancer Institute, NIH, Bethesda, MD 20892 USA; 4 Molecular Therapeutics, University of Texas M. D. Anderson Cancer Center, Houston, TX 77030 USA; 5 Department of Medicine, University of Virginia, Charlottesville, VA 22908; 6 Department of Pathology, University of Iowa Roy J. and Lucille A. Carver College of Medicine, Iowa City, IA 52242 USA

## Abstract

**Background:**

To elucidate the genes involved in the neoplastic transformation of B cells, global gene expression profiles were generated using Affymetrix U74Av2 microarrays, containing 12,488 genes, for four different groups of mouse B-cell lymphomas and six subtypes of pristane-induced mouse plasma cell tumors, three of which developed much earlier than the others.

**Results:**

Unsupervised hierarchical cluster analysis exhibited two main sub-clusters of samples: a B-cell lymphoma cluster and a plasma cell tumor cluster with subclusters reflecting mechanism of induction. This report represents the first step in using global gene expression to investigate molecular signatures related to the role of cooperating oncogenes in a model of Myc-induced carcinogenesis. Within a single subgroup, e.g., ABPCs, plasma cell tumors that contained typical T(12;15) chromosomal translocations did not display gene expression patterns distinct from those with variant T(6;15) translocations, in which the breakpoint was in the *Pvt-1 *locus, 230 kb 3' of c-*Myc*, suggesting that c-*Myc *activation was the initiating factor in both. When integrated with previously published Affymetrix array data from human multiple myelomas, the IL-6-transgenic subset of mouse plasma cell tumors clustered more closely with MM1 subsets of human myelomas, slow-appearing plasma cell tumors clustered together with MM2, while plasma cell tumors accelerated by v-Abl clustered with the more aggressive MM3-MM4 myeloma subsets. Slow-appearing plasma cell tumors expressed *Socs1 *and *Socs2 *but v-*Abl*-accelerated plasma cell tumors expressed 4–5 times as much. Both v-*Abl*-accelerated and non-v-*Ab*l-associated tumors exhibited phosphorylated STAT 1 and 3, but only v-Abl-accelerated plasma cell tumors lost viability and STAT 1 and 3 phosphorylation when cultured in the presence of the v-Abl kinase inhibitor, STI-571. These data suggest that the Jak/Stat pathway was critical in the transformation acceleration by v-Abl and that v-Abl activity remained essential throughout the life of the tumors, not just in their acceleration. A different pathway appears to predominate in the more slowly arising plasma cell tumors.

**Conclusion:**

Gene expression profiling differentiates not only B-cell lymphomas from plasma cell tumors but also distinguishes slow from accelerated plasma cell tumors. These data and those obtained from the sensitivity of v-Abl-accelerated plasma cell tumors and their phosphorylated STAT proteins indicate that these similar tumors utilize different signaling pathways but share a common initiating genetic lesion, a c-*Myc*-activating chromosome translocation.

## Background

**Figure 1:**
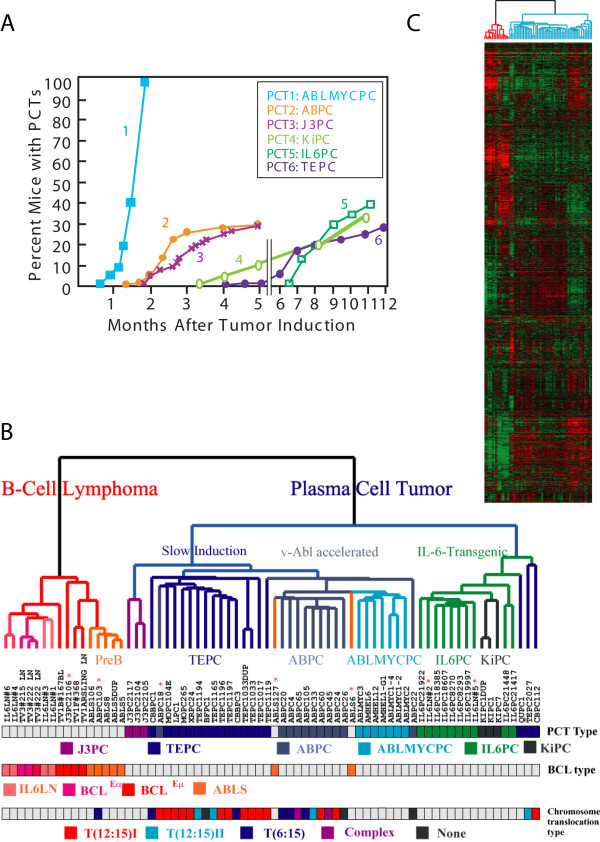
**Gene expression analysis of mouse plasmacytomas and B cell lymphomas**. 70 RNA samples from murine B-cell malignancies, comprised of 6 different types of mouse plasma cell tumors and 4 different types of mouse B cell lymphomas, were used for global gene expression studies. **A. Kinetics of appearance of 6 different subclasses of plasma cell tumors**. Time course of appearance of PCTs after ip injection of pristane. IL6PCs arose spontaneously in lymph nodes of IL-6-transgenic mice, without pristane treatment, as the mice aged. See Table 1 for more details. **B and C. Unsupervised hierarchical clustering**. Using Affymetrix U74A v2 microarrays, 6424 genes were used for clustering of genes and samples after filtering out genes with more than 50% of A (absence) calls. Correlation-based (uncentered) average linkage clustering was performed on log base 2-transformed data previously centered to mean expression values of each gene. Gene expressions above this mean value are colored red; those expressed below are shown in green. The cluster dendrogram shows 2 major sample clusters: a PCT cluster and a B-cell lymphoma cluster. Samples are coded with 11 different colors based on previously assigned groups. Asterisks indicate samples that clustered in an unexpected manner (see text). In the dendrogram, the PCT samples are colored blue-green and the BCL samples are red-orange. In the first color bar, different viral and transgenic contributions to PCT development are distinguished by different colors. In the second color bar, the 4 types of BCLs are color-coded. In the third color bar, mouse PCTs previously characterized for the fine structure of their *Myc*-activating chromosomal translocations are identified in this dendrogram. PCT samples having the variant chromosomal translocation T(6;15) are shown in deep blue, type I T(12;15) are shown in red/orange, and type II T(12;15) are shown in light blue. PCT samples having complex chromosomal translocations [12] are shown in purple, and PCT samples having no identifiable chromosomal translocations are shown in pale black. Boxes without color indicate PCTs with unknown karyotypes.

Since the discovery that BALB/c mice predictably develop plasma cell tumors (PCTs) following the induction of intraperitoneal (ip) inflammatory granulomas by implanting plastic objects or injecting mineral oil or silicones, this model has been a valuable experimental system for studying plasma cell neoplasia [1]. The ip inflammatory tissue produces interleukin 6 (IL-6), insulin-like growth factor 1 (IGF1), and other cytokines that support the growth of TEPC-type PCTs that appear slowly, starting no earlier than 120 days after ip pristane injection. Infection with retroviruses containing oncogenes, e.g., Abelson Virus (v-*Abl *[2]), J3V1 virus (v-*Raf *plus v-*Myc *[3]), RIM virus (v-Ha-*Ras *plus c-*Myc *[4]), ABLMYC Virus (v-*Abl *plus c-*Myc *[5]), accelerates PCT formation, producing ABPC, J3PCs, RIMPCs and ABLMYCPCs, respectively, in as little as 12 days (see Figure 1A and Table 1). It has never been tested whether continued activity of these accelerating oncogenes was required subsequent to completion of transformation. iMyc^Eμ ^(Myc/IgH-knock-in) BALB/c mice and IL-6-transgenic BALB/c mice also develop intraperitoneal PCTs iMyc^Eμ ^PCs [6] and KiPCs [7], respectively, at a slightly accelerated rate following pristane injection. IL-6-transgenic BALB/c mice spontaneously develop PCTs in lymph nodes (IL6PCs [8]) but with latent periods no shorter than TEPCs. As with human multiple myeloma (MM), most of the tumors in the different groups of PCTs consist of neoplasms of plasma cells that are morphologically indistinguishable, although the cells in the accelerated tumors occasionally appear larger and more blastic. Other forms of B-cell lymphomas (BCL) also appear in the lymph nodes of iMyc mice, BCL^Eμ ^[9] and BCL^Cα ^[10].

**Table 1:** Summary of mouse tumors studied by Affymetrix microarray profiling

**Tumors**	**Cited as**	**Exogenous gene effect**	**Tumor type**	**Site of origin**	**N**
PCT-1	ABLMYCPC	v-*Abl *+ c-*Myc*	Virus-accelerated ip PCT	intraperitoneal	7
PCT-2	ABPC	v-*Abl*	Virus-accelerated ip PCT	intraperitoneal	12
PCT-3	J3PC	v-*Raf *+ v-*Myc*	Virus-accelerated ip PCT	intraperitoneal	4
PCT-4	KiPC	IL-6 transgene	Transgene-accelerated ip PCT	intraperitoneal	3
PCT-5	IL6PC	IL-6 transgene	Spontaneous PCT	gut-associated lymphoid tissue	8
PCT-6	TEPC, LPC, MOPC	none	Pristane oil-induced ip PCT	intraperitoneal	18
BCL-1	IL6LN	IL-6 transgene	Spontaneous pre-malignant PCT	gut-associated lymphoid tissue	6
BCL-2	Pre B v-Abl (a.k.a. ABLS)	v-*Abl*	Virus-accelerated Pre-B lymphoma	bone marrow + spleen	6
BCL-3	BCL^Eμ^	c-*Myc *in *Igμ *locus	Spontaneous BCL	lymph nodes	3
BCL-4	BCL^Cα^	c-*Myc *in *Igα *locus	Spontaneous BCL	lymph nodes	3

Chromosomal translocations that deregulate expression of the proto-oncogene c-*Myc *constitute the earliest known tumorigenic step in PCT formation. More than 95% of PCTs, have chromosomal translocations, either the typical (85% of PCTs) T(12;15) or variants (15% of PCTs) T(6;15) or T(15;16) [11-13]. T(12;15) joins the immunoglobulin heavy chain locus and its strong enhancers to different regions: within the c-*Myc *locus (Class I) or upstream of it (Class II). T(6;15) joins the immunoglobulin kappa light chain locus with its enhancers to a site in the *Pvt1 *locus, between 200 and 300 kb 3' of the c-*Myc *locus [1,11]. *Myc *activation appears to be the hallmark of all typical BALB/c PCTs, presumably via Ig enhancer insertion near c-*Myc*. It has been assumed that the variant translocations also contribute to the transformation of plasma cells by upregulating c-*Myc *by some mechanism akin to but different from the 12;15 translocations. This notion has never been experimentally validated.

To investigate the mechanisms underlying *Myc*-induced B-cell neoplasia (particularly PCTs) in BALB/c mice, gene expression profiles for four groups of BCLs and for PCTs from six different induction protocols (Table 1) were generated. The analysis of the microarray gene expression data focused on four questions: 1) which genes differentiate BCLs from PCTs; 2) which genes and pathways are involved in PCT acceleration by the v-*Abl *oncogene; 3) are there genes that differ between PCTs induced by typical and variant c-*Myc*-activating chromosomal translocations; 4) how much similarity in gene expression profiles exists between mouse PCTs and human MMs? To answer the final question, a meta-analysis was performed by combining our mouse tumor expression data with data published for human multiple myelomas to determine similarities and differences between these plasma cell tumors in different species.

## Results

### Unsupervised clustering yielded two main clusters: B cell lymphoma cluster and plasma cell tumor cluster

To assess the relative similarities or dissimilarities in global gene expression patterns from six different subtypes of mouse PCT (PCT-1 – PCT-6, Table 1) and four different groups of mouse BCL (BCL-1 – BCL-4, Table 1), we first applied unsupervised hierarchical cluster analysis using correlation-based average linkage clustering. Characteristic expression signatures were searched for among the 6424 genes remaining after filtering out genes showing more than 50% absence (A) calls among the 70 RNA samples. This analysis revealed two principal groups, composed of BCLs and PCTs, as shown in Figures 1B and 1C.

With few exceptions, most of the PCTs, regardless of transgenic differences or viral agents accelerating appearance of PCTs, clustered together (blue-green dendrogram, Figures 1B and 1C). This cluster included both forms of PCTs that arose in IL-6-transgenic BALB/c mice: KiPCs, which arose intraperitoneally after ip pristane, and IL6PCs, which spontaneously arose in hyperplastic lymph nodes, independent of ip pristane. These results indicated that all PCTs share a common genetic expression signature. The IL-6-pre-malignant transgenic lymph node samples (IL6LN) did not cluster with the IL6PCs. Instead, these clustered with the BCLs (red-orange dendrogram, Figures 1B and 1C). ABLS are pre-B cell lymphomas induced by Abelson virus infection. Note that while the v-*Abl*-containing retroviruses also produced two subtypes of PCTs (ABPCs and ABLMYCPCs), the ABLSs clustered with BCL^Eμ ^and BCL^Cα^, suggesting that cell type influenced gene expression more than v-Abl, in this situation.

The nature of c-*Myc*-activating chromosomal translocations, i.e., typical T(12;15) vs. variant T(6;15) [11,12], did not affect their inclusion in the PCT group, nor did it lead to subclusters within the ABPC or TEPC subgroups in this particular analysis (Figure 1B and 1C). Instead, tumor sample subclustering was influenced more by the agents used to accelerate PCT development, generating subclusters within the PCT cluster, each of which contained virtually only one PCT subclass, ABLMYCPC, ABPC, IL6PC, KiPC or TEPC. Note that ABPCs and ABLMYCPCs clustered with each other before clustering with other PCTs, indicating a greater degree of similarity between these two groups of PCTs, both of which were accelerated by v-*Abl*. The two forms of PCTs that arose in IL-6-transgenic BALB/c mice also clustered most closely with one another.

Constitutive expression of IL-6 in IL-6-transgenic mice triggers polyclonal plasmacytosis in lymphoid organs such as lymph nodes and spleen [14], and neoplastic transformation of these plasma cells occurs spontaneously over time [13]. Thus, RNA samples from 6 enlarged lymph nodes in young IL-6-transgenic BALB/c mice were provisionally designated IL6LN (rich in pre-malignant cells), while samples from 8 larger lymph nodes from older mice were designated IL6PC (now containing transformed plasma cells). As expected, hierarchical clustering grouped all 8 IL6PCs into the "Plasma Cell Tumor" cluster. However, two of the "IL6LN" samples, expected to be pre-malignant, owing to the younger ages of these transgenic mice, clustered with the IL6PC samples in the PCT group and are marked with asterisks in Figure 1B. Since the remaining four samples of IL6LN were tightly clustered with the bulk of the B-cell lymphomas in the BCL group, we took this finding as evidence that the two PCT-like IL6LN samples probably contained a significant percentage of transformed plasma cells.

**Figure 2:**
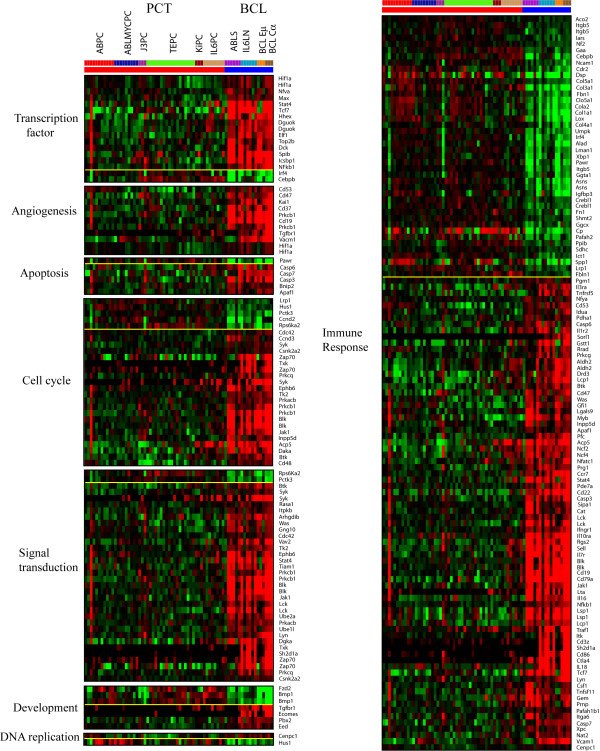
**Genes showing significant differences in expression between PCTs and BCLs**. Class comparison between PCTs and BCLs showed 926 genes that showed significant differences (p < 1 × 10^-5^) in two sample t-test. The genes assigned to 8 different functional categories by CGAP are presented as a heat map of gene cluster. Additional details can be found in Additional file 1.

Class comparison of the two main clusters revealed major, consistent gene expression differences between PCTs and BCLs. To gain biological insight into these differences, a two-sample t-test was applied to select the genes whose expression was significantly different between these two groups (926 genes, p < 1 × 10^-5^). Functional enrichment analysis [see Additional file 1] based on gene ontology (GO) terms showed that these expression differences were heavily enriched for B cell-specific genes that were generally downregulated in plasma cells [15-17]. Figure 2 presents heat maps of a subset of those genes from categories of gene functions assigned by Cancer Genome Anatomy Project (CGAP), illustrating that that PCTs and BCLs mobilize different signal transduction pathways. It is interesting to note that the majority of the gene differences consist of downregulation of genes in PCTs. It is likely that this reflects the wholesale focusing of the protein synthesis machinery of these antibody-secreting "factories" on the production of their characteristic protein.

The genes with higher expression in PCTs include Cyclin D2 (Ccnd2), syndecan1 (Sdc1), Irf4, and Xbp1. Irf4 and syndecan1 (CD138) were previously reported to have increased expression in plasma cells [15,16,18-20]. Cyclin D2 was previously reported to be induced by c-*Myc *activation [21]. Several pro-collagens (Col1a1, Col1a2, Col3a1, and Col5a1) also showed higher expression in PCTs than in BCLs. As expected, transcription factors previously known to have higher expression in plasma cells, Xbp1, Cebpb, and Irf4 [15,16] also showed higher expression in PCT groups.

B-cell surface markers (e.g., CD79b, CD19, and CD22) were highly expressed in BCL samples, as expected. Expression of caspases (e.g., Casp3, Casp6 and Casp7) was higher in the BCL group, as was expression of the chemokines (Ccr7), cytokines (IL-16, IL18) and cytokine receptors (IL1r2, IL3ra, IL7r, and IL10ra). An additional relevant gene that was more highly expressed in the BCL group was Syk, a tyrosine kinase that is essential for B-cell antigen receptor triggering of cellular activation [22]. Genes involved in Jak-Stat signaling (Jak1, Stat4) and NFκB signaling (NFκB1) also showed higher expression in the BCL groups. None of these functions are thought to be active after B cells differentiate into plasma cells, which have lost nearly all their surface receptors and downregulated many other B-cell-specific genes. Although whole lymph nodes were used as samples in IL-6-transgenic PCTs and LN, the lower expression of B-cell surface markers seen in the expression profiles of the IL6PCs suggests that the main populations of cells in IL-6-transgenic mouse lymph nodes are plasma cells, which typically lose expression of most surface markers. Our results confirm the histological finding that percentage of B- and T lymphocytes decreased as the lymph nodes fill with plasma cells and PCTs [14].

### Genes reflecting v-Abl-accelerated PCT induction

A direct comparison between two groups of PCTs: the rapid onset PCTs (ABPC and ABLMYC) and the slow onset PCTs, (TEPC, KiPC and IL6PC) was performed to get information on genes that might have contributed to the acceleration. A two-sample t-test between these two groups revealed 1195 genes with significant (p < 0.001) expression differences. Among these 1195 genes, 80 genes showed greater than 2-fold higher expression in the ABPC plus ABLMYC group, while 83 genes showed greater than 2-fold higher expression in the TEPC, KiPC and IL6PC group [see Additional file 2]. Several genes involved in cellular growth and proliferation (Cdkn2b, Tgfb1, Ptprc, Cd6, Gfi1, and Mybl2) showed higher expression in the ABPC plus ABLMYC group, as did many interferon-induced genes, presumably induced by the presence of retroviruses in this group. Several genes involved in cell death (Ada, Bid1, Gas6, Prkcζ,) were higher in TEPCs, KiPCs and IL6PCs. Table 2 lists a subset of these genes arranged by their categories of gene functions assigned by CGAP.

**Table 2:** Genes showing significant differences in expression between rapid-forming PCTs (ABPC and ABLMYCPC) and slow-forming PCTs (TEPC, IL6PC and KiPC)

***Higher in slow-forming PCT (TEPC, IL6PC, KiPC***							
**Gene function**	**Rapid PCT**	**Slow PCT**	**Fold difference (slow/rapid)**	**Description**	**Unigene ID**	**Gene symbol**	**p-value**

**Angiogenesis**							

	308.4	495.6	1.6	Neogenin	Mm.42249	Neo1	1.05E-05

**Apoptosis**							

	2872.8	5249.1	1.8	RIKEN cDNA A630035D09 gene	Mm.238213	Bcl2l1	4.67E-05

**Cell cycle**							

	583.6	2799.1	4.8	Megakaryocyte-associated tyrosine kinase	Mm.2918	Matk	< 1E-07
	270.1	912.6	3.4	Protein kinase C, zeta	Mm.28561	Prkcz	< 1E-07
	208.1	364.3	1.8	Acid phosphatase, prostate	Mm.19941	Acpp	2.10E-06
	280.7	473.5	1.7	Cyclin D1	Mm.273049	Ccnd1	7.12E-05
	1484.3	2441.1	1.6	Casein kinase 1, epsilon	Mm.30199	Csnk1e	3.50E-06

**Immune response**							

	204.1	535.4	2.6	Prolactin receptor	Mm.10516	Prlr	5.4E-06
	124	267.7	2.2	Transforming growth factor, beta receptor III	Mm.200775	Tgfbr3	1.2E-06
	2872.8	5249.1	1.8	RIKEN cDNA A630035D09 gene	Mm.238213	Bcl2l1	4.67E-05
	707.2	1243.1	1.8	AU RNA binding protein/enoyl-coenzyme A hydratase	Mm.252034	Auh	3.2E-06
	1000.1	1751.7	1.8	Aldehyde dehydrogenase 2, mitochondrial	Mm.284446	Aldh2	3.56E-05
	2990.5	5228.6	1.7	Glucosidase, beta, acid	Mm.5031	Gba	< 1E-07
	942.5	1629.7	1.7	AU RNA binding protein/enoyl-coenzyme A hydratase	Mm.252034	Auh	< 1E-07
	7349	12562.5	1.7	CCAAT/enhancer binding protein (C/EBP), beta	Mm.347406	Cebpb	1E-07
	752.1	1285.1	1.7	Profilin 1	Mm.2647	Pfn1	3.5E-06
	912.2	1538.3	1.7	Aldehyde dehydrogenase 2, mitochondrial	Mm.284446	Aldh2	6.2E-05
	2311.2	3862.5	1.7	Epidermal growth factor receptor pathway substrate 15	Mm.318250	Eps15	1.24E-05
	166.2	274.5	1.7	Microphthalmia-associated transcription factor	Mm.333284	Mitf	7.9E-06
	722.3	1097.6	1.5	Amyloid beta (A4) precursor protein-binding, family B, member 1	Mm.38469	Apbb1	7E-06
	749.6	1135	1.5	Glutathione synthetase	Mm.252316	Gss	7.61E-05

**Transcription factor**							

	294	803.5	2.7	AT motif binding factor 1	Mm.196564	Atbf1	< 1e-07
	247.9	639.4	2.6	E74-like factor 3	Mm.3963	Elf3	6.80E-06
	844.6	1883	2.2	Pre B-cell leukemia transcription factor 1	Mm.43358	Pbx1	< 1E-07
	765.9	1554.6	2.0	POU domain, class 2, transcription factor 2	Mm.208700	Pou2f2	2.30E-06
	7349	12562.5	1.7	CCAAT/enhancer binding protein (C/EBP), beta	Mm.347406	Cebpb	1.00E-07
	162.2	252.2	1.6	RIKEN cDNA 2310011G17 gene	Mm.254233	2310011G17 Rik	6.55E-05

**Signal transduction**							

	583.6	2799.1	4.8	Megakaryocyte-associated tyrosine kinase	Mm.2918	Matk	< 1E-07
	270.1	912.6	3.4	Protein kinase C, zeta	Mm.28561	Prkcz	< 1E-07
	1484.3	2441.1	1.6	Casein kinase 1, epsilon	Mm.30199	Csnk1e	3.50E-06
	456.5	729.4	1.6	Homeodomain interacting protein kinase 3	Mm.257925	Hipk3	2.11E-05

**Development**							

	844.6	1883	2.2	Pre B-cell leukemia transcription factor 1	Mm.43358	Pbx1	< 1E-07

***Higher in rapid-forming PCT (ABPC and ABLMYCPC)***							

**Gene function**	**Rapid PCT**	**Slow PCT**	**Fold difference (rapid/slow)**	**Description**	**Unigene ID**	**Gene symbol**	**p-value**

**Angiogenesis**							

	1868.3	902.8	2.1	Plasminogen activator, urokinase	Mm.4183	Plau	8.29E-05
	9821.9	4360.2	2.3	CD53 antigen	Mm.316861	Cd53	3.40E-06

**Apoptosis**							

	2588.3	1681.2	1.5	BCL2-antagonist/killer 1	Mm.2443	Bak1	9.11E-05

**Cell cycle**							

	2231.6	1126.6	2.0	Proliferation-associated 2G4	Mm.4742	Pa2g4	2.76E-05
	4858.7	2552.3	1.9	Adenylate kinase 2	Mm.29460	Ak2	< 1E-07
	10477	6082.1	1.7	CDC28 protein kinase regulatory subunit 2	Mm.222228	Cks2	< 1E-07
	1371.5	869.3	1.6	Casein kinase II, alpha 2, polypeptide	Mm.51136	Csnk2a2	1.90E-06

**Immune response**							

	3568.1	386	9.2	CD6 antigen	Mm.290897	Cd6	2.00E-07
	984.7	186.1	5.3	Tubulin, alpha 3	Mm.287784	Tuba3	< 1E-07
	2661.9	718.2	3.7	BH3 interacting domain death agonist	Mm.235081	Bid	< 1E-07
	7492.6	2554.5	2.9	Acetyl-Coenzyme A dehydrogenase, medium chain	Mm.10530	Acadm	< 1E-07
	3195.6	1346.2	2.4	Inositol (myo)-1(or 4)-monophosphatase 1	Mm.183042	Impa1	< 1E-07
	2455	1064.3	2.3	RAS-related protein-1a	Mm.333868	Rap1a	< 1E-07
	9821.9	4360.2	2.3	CD53 antigen	Mm.316861	Cd53	3.40E-06
	7479.7	3365.9	2.2	Transporter 1, ATP-binding cassette, sub-family B (MDR/TAP)	Mm.207996	Tap1	< 1E-07
	7347.8	3535.9	2.1	Superoxide dismutase 1, soluble	Mm.276325	Sod1	< 1E-07
	365.9	179.9	2.0	Growth factor independent 1	Mm.2078	Gfi1	1.30E-05
	2231.6	1126.6	2.0	Proliferation-associated 2G4	Mm.4742	Pa2g4	2.76E-05
	1939.9	985.5	2.0	RAS-related protein-1a	Mm.333868	Rap1a	4.00E-07
	450.3	261.9	1.7	Ets variant gene 4 (E1A enhancer binding protein, E1AF)	Mm.5025	Etv4	1.40E-06
	3031.7	1830.6	1.7	Wiskott-Aldrich syndrome homolog (human)	Mm.4735	Was	5.13E-06
	3136.7	1944.6	1.6	Chromosome segregation 1-like (S. cerevisiae)	Mm.22417	Cse1l	2.67E-05
	5738.7	3586.7	1.6	Survival motor neuron 1	Mm.2025	Smn1	2.75E-05
	395.2	252.8	1.6	Dihydrofolate reductase	Mm.23695	Dhfr	2.70E-06
	2017.5	1297.8	1.6	FK506 binding protein 12-rapamycin associated protein 1	Mm.21158	Frap1	8.46E-05
	2588.3	1681.2	1.5	BCL2-antagonist/killer 1	Mm.2443	Bak1	9.11E-05
	4410.1	2873.4	1.5	Ribonucleotide reductase M1	Mm.197486	Rrm1	5.14E-05
	5971.6	3940.2	1.5	Integrin beta 4 binding protein	Mm.271674	Itgb4bp	6.94E-05
	18860.1	12494.5	1.5	Triosephosphate isomerase 1	Mm.4222	Tpi1	8.30E-06

**Transcription factor**							

	6231.6	1885.8	3.3	Lymphoblastomic leukemia	Mm.4925	Lyl1	< 1E-07
	3274.2	1544.3	2.1	Chromodomain helicase DNA binding protein 1	Mm.8137	Chd1	< 1E-07
	450.3	261.9	1.7	Ets variant gene 4 (E1A enhancer binding protein, E1AF)	Mm.5025	Etv4	1.40E-06
	3136.7	1944.6	1.6	Chromosome segregation 1-like (S. cerevisiae)	Mm.22417	Cse1l	2.67E-05

**Signal transduction**							

	4858.7	2552.3	1.9	Adenylate kinase 2	Mm.29460	Ak2	< 1E-07
	10477	6082.1	1.7	CDC28 protein kinase regulatory subunit 2	Mm.222228	Cks2	< 1E-07
	3031.7	1830.6	1.7	Wiskott-Aldrich syndrome homolog (human)	Mm.4735	Was	5.13E-05
	1371.5	869.3	1.6	Casein kinase II, alpha 2, polypeptide	Mm.51136	Csnk2a2	1.90E-06

**DNA replication**							

	3805.2	2420.4	1.6	Small nuclear ribonucleoprotein polypeptide A'	Mm.821	Snrpa1	< 1e-07
	8247.5	4325.9	1.9	Replication protein A1	Mm.180734	Rpa1	4.20E-06

Class comparison between slow-forming PCTs (TEPC, IL6PC and KiPC) and rapid-forming PCTs (ABPC and ABLMYCPC) by two-sample t-test generated 1195 genes showing significant differences (p- value < 0.001). Among these 1195 genes, the genes showing more than 1.5 fold increase and p-value < 0.0001 were selected in this table. The genes in each category of gene function were assigned based on the annotation of CGAP.

### Effects of v-Abl

The gene expression levels of *Socs1 *and *Socs2 *(Suppressors of Cell Signaling 1 and 2) showed very high levels in all Abelson virus-infected samples (ABPC, ABLMYCPC) compared to other PCTs [see Additional file 2]. Socs 1 & 2 are proteins that normally interrupt Jak/Stat signaling [23], but their growth-inhibiting effects are abrogated by the v-Abl protein [24,25]. These high levels of Socs expression are likely to reflect the continuous activation of Jak-STAT expected from v-Abl [26]. This hyperactive Jak/STAT pathway may be partly responsible for the rapid appearance of ABPCs and ABLMYCPCs compared to TEPCs, as shown in Figure 1A.

### Sensitivity of v-Abl accelerated PCTs to treatment with STI-571

**Figure 3:**
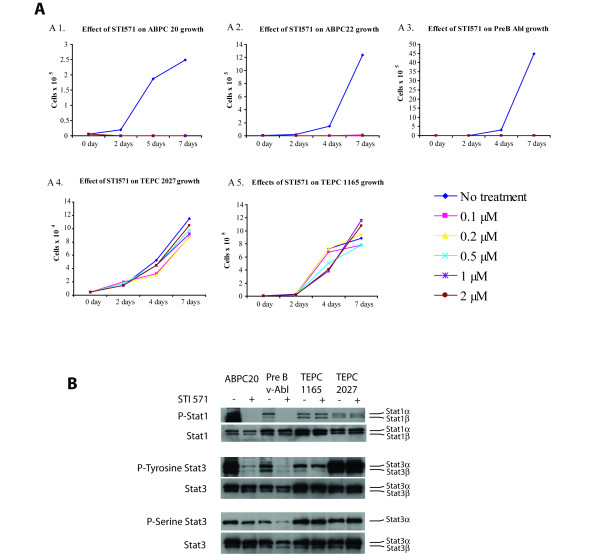
**Greater sensitivity of ABPCs to STI-571 than TEPCs. A. Cell proliferation analysis of PCTs after treatment with STI-571**. Five different cell lines including two ABPCs (ABPC20, ABPC22), two TEPCs (TEPC1165, TEPC 2027) and one pre B v-Abl lymphoma were treated with different concentrations of STI-571 at an initial concentration of 5 × 10^3 ^cells/well. Cell numbers were measured at 4 different times (0, 2, 4, and 7 days after treatment). **B. Western blot analysis of PCTs for STAT1 phosphorylation and STAT3 phosphorylation**. Samples for western blot were collected from untreated cells and cells after treatment with 5 μM STI-571 for 18 hours. Doublet bands are the α and β forms of the STAT proteins.

The main difference between ABPCs and TEPCs is the acceleration of plasma cell tumor formation by the v-*Abl *gene. The high levels of *Socs1 *&*2 *expression in ABPCs suggested that v-Abl was active in the fully mature PCTs. To confirm this finding and to determine whether fully transformed PCTs still retained a requirement for the v-Abl kinase that accelerated its appearance, cell survival analysis was performed after treatment of tissue culture lines of ABPC 20, ABPC 22, TEPC 1165, TEPC 2027 and pre B v-Abl lymphoma with 0.1 to 2.0 μM STI-571, an Abl kinase inhibitor [27]. Figure 3A shows a clear difference in the susceptibility of those cell lines to STI-571. ABPCs (ABPC20, ABPC22) and pre B cell lymphoma (pre B v-Abl) generated by Abelson virus infection showed higher susceptibility to STI-571 with complete inhibition of cell growth at 0.1 μM of STI-571 solution, while TEPC 1165 and TEPC 2027 showed no inhibition of cell growth even at 2 μM STI-571. Those data suggest that v-*Abl *is still required for cell survival and proliferation even in fully transformed ABPCs as was previously shown for v-*Abl*-induced pre B lymphomas [28].

### STI-571 inhibits Stat activation of v-Abl accelerated PCT and pre B lymphoma

The preceding analysis suggests that the v-*Abl*-induced PCTs utilize the Jak/STAT signaling pathways. As shown in Figure 3B, the PCT cell lines studied showed highly activated (phosphorylated) STAT1 and STAT3. However treatment with 5 mM STI-571 led to decreased phosphorylation of STAT1 and STAT3, but only in the cell lines induced with Abelson virus, ABPC 20 cell line and pre B v-Abl lymphoma. This dose of STI-571 had no effect on phosphorylation of STAT1 or STAT3 in TEPC 1165 and TEPC 2027. Presumably the latter PCTs also utilized the Jak/Stat signaling pathways, but they were not stimulated by Abl. What is more, 5 μM STI-571 completely inhibited the STAT1 phosphorylation in ABPC 20 and pre B v-Abl lymphoma and the STAT3 tyrosine phosphorylation in pre B v-Abl, and it virtually completely inhibited the tyrosine and serine phosphorylation of STAT3 in ABPC 20. Signals activating STAT3 can also come from other different pathways (e.g., those responsible for STAT phosphorylation in TEPC 1165 and TEPC 2027, such as Ras activation by c-Myc). In contrast, STAT3 activation of the pre B v-Abl line comes almost exclusively from v-Abl, as was shown in v-Abl-transformed pre B-cells [24]. STI-571's selective inhibition of Stat activation in ABPC 20 and pre B v-Abl lymphoma suggests that the v-Abl signaling pathways are still critical for viability, even in fully transformed tissue-cultured tumors.

### Quantitative RT-PCR analysis

**Figure 4:**
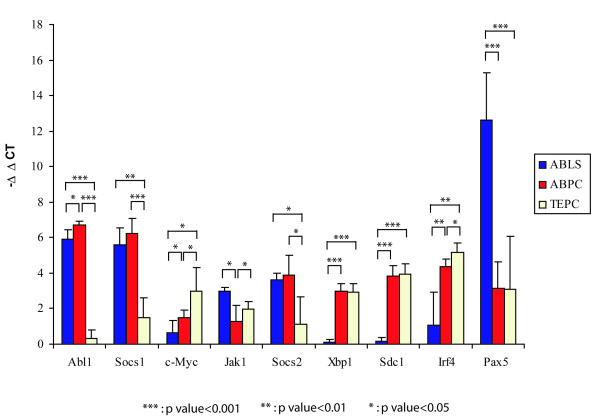
**Quantitative real-time PCR analysis**. qRT-PCR was performed on RNAs from 3 different groups of cultured cells to analyze relative mRNA content for 9 key genes: c-*Myc, Socs1*, *Socs 2, Abl1, Jak1, Xbp1, Sdc1, Irf4, Pax5 *as well as the "housekeeping gene", GAPDH, using primers and detection oligos designed in consultation with the manufacturer (ABI). The data was presented as mean ± standard deviation values of each group. qRT-PCR data for individual samples are presented as Additional file 3. All data were normalized to GAPDH. These values were then calibrated against the sample with the lowest expression and represented as -ΔΔCt values with standard errors.

For validation of certain key findings in the microarray data, real time RT-PCR was performed for 9 selected genes that may play significant roles in PCTs (Figure 4). Thirteen different tumor RNA samples (6 ABPC, 4 TEPC and 3 ABLS) were analyzed, using RNA from the sample that showed the lowest expression as calibrator for each gene. Means for each of the three tumor types are shown in Figure 4, and individual results are presented in Additional file 3. These results paralleled the mRNA expression levels acquired via microarray analysis. c-*Myc *expression was generally highest in the four TEPC samples, while it showed lowest expression in the v-*Abl*-expressing ABLS (pre-B cell lymphomas). Socs1 and Socs2 expression was higher in ABPC and ABLS than in TEPC samples. The genes previously known to be plasma cell-specific transcription factors (Xbp1 and Irf4) and surface marker (Sdc1/CD138) showed higher expression in PCTs than in pre-B cell lymphomas (ABLS). Jak1 expression was higher in the pre-B lymphomas than in the PCTs.

### Differences of gene expressions based on c-Myc gene chromosomal translocations

Cluster analysis suggested that the site of chromosomal translocation on chromosome 15 and the nature of its translocation partner chromosome did not affect the gene expression profile as much as additional oncogenes play in PCT induction. To test this concept more stringently, a two-sample t-test was used to search for differences in gene expression between typical T(12;15) and variant T(6;15) chromosomal translocations within a single subgroup of PCTs, ABPCs. Only 14 genes, most of which had low levels of expression, showed significant difference in expression, suggesting that the site of translocation had little effect on the mechanism of transformation of the PCTs [see Additional file 4]. Another t-test was performed to compare the effect of two different forms of T(12;15), class I or II, in TEPCs [see Additional file 4]. This showed 29 genes with significant differences in expression, also relatively low in expression, consistent with the previous comparison and our interpretation that the different forms of chromosomal translocation did not affect significant differences in mechanism of transformations or the type of cell being transformed. This analysis cannot rule out some differences in early neoplastic events, since we were only examining expression values from fully transformed tumors.

### Meta-analysis of the mouse expression data co-mingled with expression pattern data for human plasma cell neoplasms

**Figure 5:**
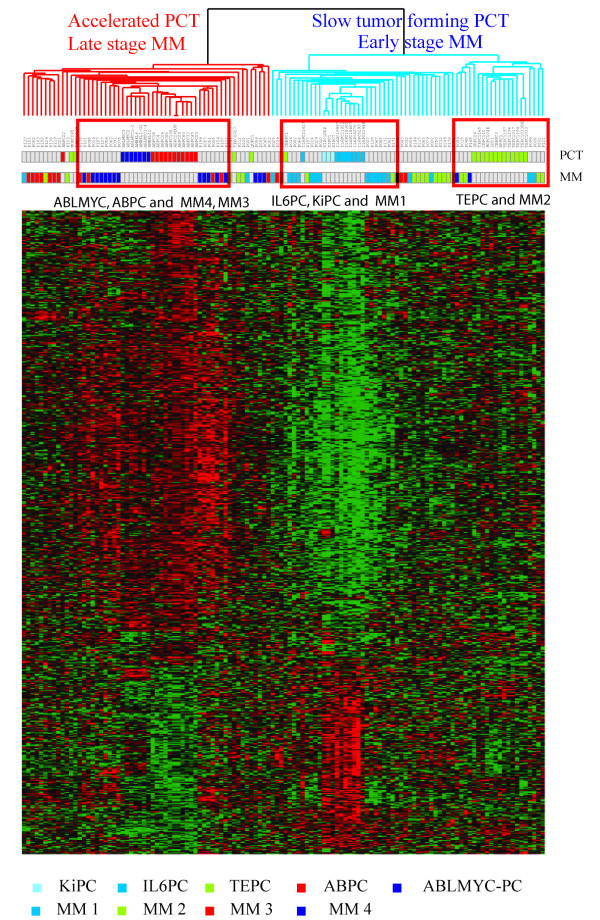
**Meta-analysis of human multiple myeloma samples with mouse plasmacytomas**. A total of 122 samples, comprised of 74 human samples and 48 mouse samples, were used for this meta-analysis. Human samples had previously classified into 4 different stages of multiple myeloma (MM1, MM2, MM3 and MM4, generally reflecting increasing severity or stage of disease [18]), and mouse samples were composed of 5 different PCT groups, ABPC and ABLMYCPC, "two accelerated subgroups [PCT-1 and -2] (Table 1)", and "TEPC, KiPC and IL6PC, the slower appearing subgroups [PCTs 4 – 6] (Table 1)". The stage of myeloma and group of plasma cell tumors to which each sample belongs is indicated by the color of the rows of boxes beneath sample names, with a color key beneath the heat map. The human and mouse data were normalized and standardized separately and then combined for analysis [40,41]. The genes for cluster analysis were selected by one-way ANOVA analysis (2266 genes, p-value < 0.001) of mouse PCTs, and 634 orthologs from these 2266 genes, present both in human and mouse array platforms were used for cluster analysis.

Having defined distinctive gene expression patterns among the PCT subtypes as well as between PCTs and BCLs, we next sought to examine how well our mouse models recapitulate human phenotypes as measured by relative similarities of gene expression patterns. Mouse plasmacytomas form in the intraperitoneal cavity, while human multiple myelomas are found in bone marrow, very different locations but not without potentially meaningful similarities: richness in adipocytes, plentiful reactive myeloid cells, abundant blood supply, among others. We used gene expression data from human B lymphocytes and human multiple myeloma isolates, and cross-compared them with the gene expression data from our models. Using genes that existed as orthologues in both human and mouse Affymetrix chips, unsupervised cluster analysis showed that BCLs (human and mouse) clustered separately from the PCTs and MMs [see Additional file 5]. Since the B-cell components in this series were small in number, we focused our analysis on the plasma cell components, human and murine. The human multiple myeloma samples had been previously assigned to MM1-MM4 subgroups, based on cluster analysis of gene expression patterns of bone-marrow plasma cells from patients with multiple myeloma [18,19]. When PCTs and MMs were re-analyzed without the B-cell components, there was co-stratification of mouse and human tumors in such a way that the more aggressive MM3 and MM4 co-clustered with the accelerated PCT-1 and -2 (ABLMYCPCs and ABPCs), while MM1 co-clustered with IL-6 transgenic PCTs (KiPCs and IL6PCs) (Figure 5). The slow-arising TEPCs (PCT-6) co-clustered with the less aggressive MM1-MM2 patients. This intriguing inter-species co-clustering of plasma cell neoplasms will be investigated in greater depth in another study that will incorporate more recent myeloma data [29] that present a new molecular classification of myelomas into seven subgroups.

## Discussion

Microarray expression profiling has been quite successful in differentiating subclasses within tumors with a similar histological/morphological diagnosis [18,30]. Tumors with common genetic lesions tend to cluster together, reflecting the fact that deregulation of one or more genes leads to downstream gene activation/inactivation cascades that are unique to that lesion. It has also made it possible to predict the prognosis of cancer patients and the likelihood of their responding to chemotherapy, based on gene expression patterns [18,31]. Major progress in experimental cancer research will require animal model systems and *in vitro *cell culture systems. Experimentally induced PCTs and related BCLs have long been, and continue to be, useful for the study of plasma cell neoplasms.

In this study, we generated gene expression profiles of mouse B-lymphocytic neoplasms, including four groups of PCTs that arose with different latent periods in chronic inflammatory tissue induced by ip pristane. We also studied two subgroups of PCTs that arose spontaneously in hyperplastic lymph nodes of IL-6-transgenic mice and B-cell lymphomas from i*Myc *gene-insertion mice. Statistical methods were employed to compare these expression profiles to search for specific gene expression signatures that might aid in understanding the molecular mechanisms at work in each subtype of B cell malignancy and the differences in tumor latent periods resulting from variation in the PCT induction schemes.

Classical unsupervised clustering analysis and statistical analysis of microarray data revealed a tight clustering of PCTs regardless of subtype. The expression patterns in this group showed major differences from those of the B-cell lymphoma group. As seen in earlier analyses [30], the stage of B cell differentiation strongly influenced the major gene expression differences. It is important to note that virtually all of the PCTs clustered together, including both intraperitoneal oil-induced PCTs as well as those that arose spontaneously in IL-6-transgenic lymph nodes, independent of ip pristane. These results indicated that all PCTs share a common genetic expression signature. It can also be seen that many genes present in the B-cell signature have been downregulated in the PCT signature, a well-known feature of the specialized plasma cell, which seems to reflect this cell's single-minded dedication to maximize synthesis of antibody Ig.

Many of the genes that emerged as part of the PCT signature after unsupervised clustering analysis had been recognized in previous studies of gene expression patterns as characteristic of this stage of B cell differentiation, before and after neoplastic transformation [17,19,32]. Irf4 and Syndecan1 are characteristically expressed in plasma cells, and cyclin D2 has been reported to have high expression in PCT, presumably secondary to deregulated c-*Myc*.

Subclusters within the PCT cluster were seen, chiefly reflecting the slow vs. rapid appearance of the PCTs and the nature of the accelerating oncogenes. Unlike human myelomas, mouse plasma cell tumors have a common initiating event, a Myc-activating chromosome translocation. We found that unsupervised clustering of these tumors did not reveal major differences in gene expression that could be associated with position of breakpoint and translocation on chromosome 15. That is, the nature of c-*Myc*-activating chromosomal translocations [i.e., typical T(12;15) vs. variant T(6;15)] did not affect their inclusion in the PCT group, nor did it lead to subclusters within the broad PCT grouping or the ABPC or TEPC subgroups, in this particular analysis. This supports our hypothesis that even T(6;15) translocations that occur within the Pvt-1 region initiate PCTs via induction of constitutive c-*Myc *expression, and that fine structural differences in the site of T(12;15) within or near the c-*Myc *locus itself do not materially affect the way Myc influences PCT induction. Of course, we know that additional genetic events are required for complete transformation of plasma cells, for Myc-activating chromosome translocations have been found in normal cells and organs that did not develop plasma cell tumors [33]. Future studies will focus on identifying these additional events that occur during tumor progression.

Our cluster analysis did, however, reflect differences in the cooperating genes used to accelerate tumorigenesis. The most striking gene expression changes associated with tumors accelerated by Abelson virus infection are the high levels of *Socs1 *expression. The Socs proteins are inhibitors of signaling pathways and are generally expressed at low levels in unstimulated cells. They become rapidly induced by cytokines, thereby inhibiting Jak-STAT signaling, forming a classic negative feedback loop [34]. It was previously shown that the v-*Abl *oncogene activates Jak-STAT signaling during transformation of pre-B cells in mice [24]. Although Socs-1 is highly expressed in v-*Abl*-transformed cells, it is unable to inhibit v-*Abl*-mediated Jak- STAT signaling. This is thought to be the result of one of the non-tyrosine kinase effects of v-Abl, such as phosphorylation of Socs-1 on non-tyrosine residues, leading to disruption of the interaction between Socs-1 and the elongin B/C complex and inhibition of Socs1-mediated proteasome targeting of activated Jaks [24]. STI-571/Imatinib (Gleevec, Novartis Pharma), at low concentrations, is a specific inhibitor of Abl kinase and shows effectiveness in the treatment of chronic myelogenous leukemia [27,35]. ABPC20, ABPC22 and pre B v-Abl are much more sensitive to killing by STI-571 than TEPC1165 or TEPC2027 (Figure 3A), indicating that the Abl kinase activity is still required for viability of PCTs induced with the assistance of Abelson Virus. Since nearly all ABPCs also show c-*Myc*-activating chromosome translocations, some cooperation between the signaling pathways of v-Abl and c-Myc must be responsible for the rapid transformation of ABPCs. Unexpectedly, however, this cooperation appears to be required even after full transformation is achieved and even after adaptation to growth in culture. It will be interesting to see if resistance to STI-571 develops in these cultures, as is often seen in human CML [31].

*Stat4 *is more highly expressed in the BCL group than in PCTs. However, the level of *stat3* expression does not differ significantly between BCLs and PCTs, both groups showing relatively high expression levels. *Jak1 *also showed higher expression in the BCL group compared to PCTs, but *Jak1 *is relatively highly expressed, even in PCTs. The accelerating mechanisms engaged after v-*Abl *infection seems to utilize these pathways (Figure 3B), despite the concomitant induction of the counteracting *Socs *family of genes. These pathways are currently being studied in greater depth at the translational and post-translational levels within the PCT system, following up the leads afforded by our gene expression studies and the initial phosphorylation studies shown here, with the goals of understanding the mechanisms at work.

It has been illuminating to analyze our mouse expression data in conjunction with already published Affymetrix data from human multiple myeloma. Cluster analysis showed that human MM1 clustered most closely with PCT4 and PCT5, IL6PC and KiPC, the two groups of PCTs from IL-6-transgenic mice, while the more aggressive myeloma groups, MM3-MM4, clustered more tightly with PCT1 and PCT2, ABLMYCPC and ABPC, those with appearance accelerated by *v-*Abl activity. This similarity includes differences in expression of genes associated with proliferation. This was unexpected but significant, because plasma cell neoplasms are not generally associated with rapid proliferation. Instead, increased survival or escape from apoptosis is thought to be the chief mechanism responsible for the expansion of lymphocytes or plasma cells in lymph nodes or bone marrow, respectively. This similarity brings to mind the possibility that Imatinib, the activated Abl inhibitor, might be effective in treating aggressive myeloma patients.

This co-clustering suggests that different pathways can be utilized to achieve a similar outcome, namely transformation of plasma cells. Thus, the mouse PCT model, despite its biological differences from MM, offers an experimental model for studying the details of the etiology of plasma cell neoplasms with different degrees of aggressiveness, much as seen in human myelomas. This aspect of our study will be broadened to include new data on additional myeloma patients [29] in which expression data are used to define seven subgroups that differ in their molecular characteristics. This study will be the subject of a separate manuscript.

## Conclusion

Lymphoid transformation and plasma cell tumor formation are complicated, multi-stage processes, so it is necessary to study these processes prospectively using research tools covering genome-wide changes in expression. The present study shows that gene expression profiling can differentiate B-cell lymphomas from plasma cell tumors and also distinguish slow from accelerated plasma cell tumors. These results and data obtained from the sensitivity of v-Abl-accelerated plasma cell tumors and their phosphorylated STAT proteins to the effects of STI-571 indicate that these otherwise similar tumors utilize different signaling pathways but share a common initiating genetic lesion, a c-*Myc*-activating chromosome translocation. This study of gene expression profiles of mouse B-cell lymphomas and several subclasses of plasma cell tumors provides data that offer clues for the understanding of B-cell neoplasia and plasma cell tumor formation and the interpretation of the prospective plasma cell tumor induction studies that are now under way.

## Methods

### Sample selection and RNA preparation

A total of 70 samples of RNA were prepared from transplanted mouse tissues. All solid PCT samples (except IL6PC) used for microarray hybridization had been transplanted at least once from the initial ip tumor tissue that arose following pristane injection. As summarized in Table 1, the four groups of BCLs and the six different PCT subtypes originated as follows. TEPCs (PCT-6) were obtained after ip injections of pristane in BALB/c mice [1]. ABPCs (PCT-2) and J3PCs (PCT-3) were obtained more rapidly (Figure 1A) by introducing Abelson virus [2] or J3V1 virus [3], retroviruses containing v-*Abl*, or v-*Raf-1 *and v-*Myc *genes, respectively, following the injection of pristane in BALB/c mice. ABLMYCPCs (PCT-1) originated even more rapidly in the pristane-conditioned peritoneum of BALB/c mice infected with ABLMYC virus [5], a retrovirus that expresses both v-*Abl *and c-*Myc *genes. IL6PCs (PCT-5) were obtained from hyperplastic lymph nodes that spontaneously developed in IL-6-transgenic BALB/c mice [8]. KiPCs (PCT-4) were generated by injecting pristane intraperitoneally into IL-6-transgenic BALB/c mice [7]. "Pre-malignant" IL6LN (BCL-1) were hyperplastic lymph nodes of IL-6-transgenic BALB/c mice [13]. ABLS (a.k.a. preB vAbl and BCL-2) were pre-B lymphomas that arose rapidly in lymphoid organs of Abelson virus-treated BALB/c mice [2]. BCL^Eμ ^(BCLs-3) are lymphoblastic B-cell lymphomas with a Burkitt-like morphology from lymph nodes of transgenic iMyc^Em ^mice [9]. BCL^Cα ^(BCL-4) were obtained from spleens of transgenic iMyc^Cα ^mice [10]. All mice were maintained in our conventional colony on the NIH campus under Animal Study Protocol LG-028. Total RNA was prepared by grinding pieces of excised, snap frozen and otherwise unprocessed tissues in liquid nitrogen and extraction in TRIzol (Invitrogen, Carlsbad, CA) followed by further purification on RNAeasy columns (Qiagen, Inc., Valencia, CA).

### Microarray hybridization

Affymetrix Murine Genome Set U74Av2 microarrays (Affymetrix Inc., West Sacramento, CA) were used for the hybridization of biotin-labeled cDNA probes synthesized from 5 μg of total RNA or 1 μg poly(A)+ RNA using a Superscript double-strand cDNA synthesis kit (Invitrogen, Carlsbad, CA), Bioarray High Efficiency RNA transcript labeling kit and Mg-catalyzed fragmentation kit (Enzo Biochemicals Inc., Farmingdale, NY) according to the manufacturers' instructions. Microarrays were stained with phycoerythrin-streptavidin (Molecular Probes, Carlsbad, CA), scanned with an Affymetrix GeneChip scanner and analyzed with Affymetrix Microarray Analysis Suite (MAS) version 5.0.

### Statistical analysis of microarray data

BRB ArrayTools Version 3.0 [36] was used for the analysis of the MAS 5.0 data set. A log base 2 transformation was applied to the data set before arrays were normalized. Each array was normalized using median values of gene expression over the entire array (global normalization). A median array was selected as the reference array for normalization. Class comparison analysis was performed with the two-sample t-test of BRB ArrayTools with the estimation of false discovery rate. Cluster analysis was performed with Cluster and Treeview [37]. For the cluster analysis, the log base 2 transformed data were centered to the mean values of each gene's expression. Significantly enriched components defined in Gene Ontology (GO) were determined based on hypergeometric distribution analysis [38].

### Meta-analysis of published data

The human multiple myeloma data and their classification into subclasses MM1 – MM4 was previously reported by Zhan et al. [18,19] using Affymetrix HuGeneFL Genome Array chips (Affymetrix, Inc., West Sacramento, CA). 3739 orthologues between the human FL microarray and the mouse U74Av2 microarray were identified based on curated Hu6800 orthology from Affymetrix [39]. Each data set was normalized separately and then combined together as reported earlier [40]. Before integrating the human data set with the mouse data set, the expression of each gene was standardized to a mean ± s.d. of 0 ± 1, independently in both data sets as described [41].

### Cell culture, proliferation assay and inhibition by STI-571

This laboratory has adapted a number of PCTs, including TEPC 1165, TEPC 2027, ABPC22 and ABPC 20 studied here, to *in vitro *growth in RPMI 1640 plus 10% fetal bovine serum, 10 ng/ml IL-6, penicillin and streptomycin. Inhibition of proliferation by STI-571/Imatinib/Gleevec (Novartis, Basel, Switzerland) [27,35] was determined using several different concentrations of inhibitor (from 0.1 μM to 2 μM) on an initial cell suspension of 5 × 10^3 ^cells/well of a 24-well plate. Cell proliferation was determined by counting the cells at 2 days, 4 days and 7 days after treatment with STI-571.

### Western blotting

Tissue cultured mouse PCT cells, with and without 5 μM STI-571, were harvested after 18 hours of treatment and were subjected to western blot analysis. Membranes were probed with the following antibodies: STAT1, STAT3, tyrosine 701-phosphorylated STAT1, tyrosine 705-phosphorylated STAT3 and serine 727-phosphorylated STAT3 (Cell Signaling, Beverly, MA).

### Quantitative real-time PCR

Quantitative RT-PCR was performed using the ABI 7500 real time PCR system following the manufacturer's protocol. Mouse GAPDH primer and probes were used as the normalization control in quantitative analysis. The primers and probes used in this study were purchased from Applied Biosystems (Foster City, CA), and primer sequences are available upon request. The relative mRNA expression levels are expressed as -ΔΔCt, in which ΔCt is the difference between the threshold PCR cycle (Ct) value of each experimental mRNA and that of the corresponding internal control, GAPDH. The -ΔΔCt value is the difference between the ΔCt value of each tissue and that of the lowest-expressing sample, used as calibrator for that gene [42].

## Authors' contributions

JFM and JDS conceived the project. JFM and JDO isolated the RNAs. ESP analyzed the data and wrote the manuscript. J-SL and HGW contributed additional statistical analyses. MP and SJ provided samples and interpretation. SG, HW, and FZ performed experiments, and JFM co-authored the manuscript and was responsible for project development. All authors read and approved the manuscript.

## Supplementary Material

Additional file 1 Supplementary Tables 1. Functional enrichment analysis of genes that showed significant differences in expression between plasma cell tumors and B-cell lymphomas. Supplementary Table 1A. Functional enrichment analysis according to GO Molecular Function category. Supplementary Table 1B. Functional enrichment analysis by GO Cellular Component category. Supplementary Table 1C. Functional enrichment analysis by GO Biological Process category. Supplementary Table 1D. Genes present in nine GO Molecular Function categories that were shown to be functionally enriched (see Supplementary Table 1A, above). Four tables showing lists of genes that showed significant (p < 0.005) differences in expression between plasma cell tumors and B-cell lymphomas characterized by their functional enrichment according to biological processes defined by the Gene Ontology Consortium. The enrichment of gene set was estimated by calculating the cumulated hypergeometric p values of the each biological process defined by the Gene Ontology Consortium . In Table 1D, 9 components from the Molecular Function GO category were selected to show the actual genes involved. Click here for file

Additional file 2 Supplementary Tables 2. Genes that showed significant (p < 0.001) differences in expression between rapid-forming plasma cell tumors (ABPC and ABLMYCPC) and slow-forming plasma cell tumors (TEPC, IL6PC and KiPC). Supplementary Table 2A. Genes that showed more than 2-fold higher expression in rapid-forming plasma cell tumors than in slow-forming plasma cell tumors. Supplementary Table 2B. Genes that showed more than 2-fold higher expression in slow-forming plasma cell tumors than in rapid-forming plasma cell tumors. Two tables showing lists of genes that showed significant (p < 0.001) differences in expression between rapid- and slow-forming plasma cell tumors. Click here for file

Additional file 3 Supplementary Figure 1. Quantitative RT-PCR of relative mRNA content in 13 mouse B-cell lymphomas and plasma cell tumors for 9 key genes. Results of quantitative RT-PCR validation of relative mRNA content in 13 mouse B-cell lymphomas and plasma cell tumors for 9 key genes: *c-Myc, Socs1, Socs2, Abl1, Jak1, Xbp1, Sdc1, Irf4, Pax5 (BSAP) *and the "housekeeping" gene, *GAPDH*. Click here for file

Additional file 4 Supplementary Tables 3. Genes with significant (p < 0.001) differences in gene expression associated with site of chromosomal translocation in two sample t-tests. Supplementary Table 3A. Genes that showed significant differences in expression between ABPCs with T(12;15) and ABPCs with T(6;15). Supplementary Table 3B. Genes that showed significant differences in expression between TEPCs with T(12;15 class I) and TEPC T(12;15 class II) chromosome translocations. Two tables showing lists of genes that showed significant (p < 0.001) differences in expression between similar subclasses of plasma cell tumors that differ in site of Myc-activating chromosomal translocation. Click here for file

Additional file 5 Supplementary Figure 2. Meta-analysis of global gene expression of 67 mouse samples (including 5 different subgroups of plasma cell tumors and B-cell lymphomas) combined with 123 human samples (including 5 different subgroups of multiple myelomas and tonsilar B cells). Heat map derived from a meta-analysis of a combination of expression data from human and mouse plasma cell dyscrasias. Click here for file
