# Supplementary material for: Gene expression profiling reveals different pathways related to Abl and other genes that cooperate with c-Myc in a model of plasma cell neoplasia
Source: BMC Genomics. 2007 Aug 31;8:302. doi: 10.1186/1471-2164-8-302 (PMC2040348; doi:10.1186/1471-2164-8-302)
Supplement: Additional file 1 — Supplementary Tables 1. Functional enrichment analysis of genes that showed significant differences in expression between plasma cell tumors and B-cell lymphomas. Supplementary Table 1A. Functional enrichment analysis according to GO Molecular Function category. Supplementary Table 1B. Functional enrichment analysis by GO Cellular Component category. Supplementary Table 1C. Functional enrichment analysis by GO Biological Process category. Supplementary Table 1D. Genes present in nine GO Molecular Function categories that were shown to be functionally enriched (see Supplementary Table 1A, above). Four tables showing lists of genes that showed significant (p < 0.005) differences in expression between plasma cell tumors and B-cell lymphomas characterized by their functional enrichment according to biological processes defined by the Gene Ontology Consortium. The enrichment of gene set was estimated by calculating the cumulated hypergeometric p values of the each biological process defined by the Gene Ontology Consortium . In Table 1D, 9 components from the Molecular Function GO category were selected to show the actual genes involved. [file 1471-2164-8-302-S1.doc]

**Additional file 1.**

Supplementary Tables 1. Functional enrichment analysis of genes that showed significant differences in expression between plasma cell tumors and B-cell lymphomas

The enrichment of gene set was estimated by calculating the cumulated hypergeometric p values of the each biological process defined by the Gene Ontology Consortium (www.gene-ontology.org). Gene annotation to Gene Ontology (GO) was downloaded from the NCBI (ftp://ftp.ncbi.nih.gov/gene). Statistical significance was determined with a cut-off of p < 0.005.

Supplementary Table 1A. Functional enrichment analysis according to GO Molecular Function category

| **Molecular function category** | | | | |
| --- | --- | --- | --- | --- |
| GO Id | p-value | Name | Numbers of genes in this category | Numbers of genes in the signature |
| GO:0015031 | (p=0.000000) | protein transport | 318 | 38 |
| GO:0007242 | (p=0.000001) | intracellular signaling cascade | 335 | 36 |
| GO:0007166 | (p=0.000010) | cell surface receptor linked signal transduction | 78 | 14 |
| GO:0006487 | (p=0.000040) | protein amino acid N-linked glycosylation | 10 | 5 |
| GO:0007264 | (p=0.000043) | small GTPase mediated signal transduction | 161 | 20 |
| GO:0006418 | (p=0.000049) | tRNA aminoacylation for protein translation | 39 | 9 |
| GO:0008154 | (p=0.000094) | actin polymerization and/or depolymerization | 3 | 3 |
| GO:0018346 | (p=0.000258) | protein amino acid prenylation | 8 | 4 |
| GO:0006139 | (p=0.000263) | nucleobase, nucleoside, nucleotide and nucleic acid metabolism | 21 | 6 |
| GO:0019886 | (p=0.000554) | antigen processing, exogenous antigen via MHC class II | 16 | 5 |
| GO:0050853 | (p=0.000878) | B cell receptor signaling pathway | 5 | 3 |
| GO:0045579 | (p=0.000878) | positive regulation of B cell differentiation | 5 | 3 |
| GO:0006915 | (p=0.001079) | apoptosis | 250 | 23 |
| GO:0018108 | (p=0.001317) | peptidyl-tyrosine phosphorylation | 19 | 5 |
| GO:0030333 | (p=0.001575) | antigen processing | 12 | 4 |
| GO:0048535 | (p=0.001690) | lymph node development | 20 | 5 |
| GO:0030889 | (p=0.001697) | negative regulation of B cell proliferation | 6 | 3 |
| GO:0030100 | (p=0.001697) | regulation of endocytosis | 6 | 3 |
| GO:0018279 | (p=0.002072) | protein amino acid N-linked glycosylation via asparagine | 2 | 2 |
| GO:0046641 | (p=0.002072) | positive regulation of alpha-beta T cell proliferation | 2 | 2 |
| GO:0045045 | (p=0.002072) | secretory pathway | 2 | 2 |
| GO:0030278 | (p=0.002072) | regulation of ossification | 2 | 2 |
| GO:0006423 | (p=0.002072) | cysteinyl-tRNA aminoacylation | 2 | 2 |
| GO:0006928 | (p=0.002663) | cell motility | 22 | 5 |
| GO:0045060 | (p=0.004436) | negative thymic T cell selection | 8 | 3 |

Supplementary Table 1B. Functional enrichment analysis by GO Cellular Component category

| **Cellular component** | | | | |
| --- | --- | --- | --- | --- |
| GO Id | p-value | Name | Numbers of genes in this category | Numbers of genes in the signature |
| GO:0005783 | (p=0.000000) | endoplasmic reticulum | 426 | 46 |
| GO:0042101 | (p=0.000001) | T cell receptor complex | 6 | 5 |
| GO:0005737 | (p=0.000003) | cytoplasm | 835 | 66 |
| GO:0042105 | (p=0.000052) | alpha-beta T cell receptor complex | 6 | 4 |
| GO:0009897 | (p=0.000094) | external side of plasma membrane | 86 | 13 |
| GO:0005581 | (p=0.000165) | collagen | 28 | 7 |
| GO:0001772 | (p=0.000330) | immunological synapse | 4 | 3 |
| GO:0019815 | (p=0.001542) | B cell receptor complex | 6 | 3 |
| GO:0005814 | (p=0.001542) | centriole | 6 | 3 |
| GO:0005634 | (p=0.001878) | nucleus | 2693 | 148 |
| GO:0008250 | (p=0.001939) | oligosaccharyl transferase complex | 2 | 2 |
| GO:0015630 | (p=0.001943) | microtubule cytoskeleton | 13 | 4 |
| GO:0005764 | (p=0.002560) | lysosome | 93 | 11 |
| GO:0008290 | (p=0.004039) | F-actin capping protein complex | 8 | 3 |

Supplementary Table 1C. Functional enrichment analysis by GO Biological Process category

| **Biological Process** | | | | |
| --- | --- | --- | --- | --- |
| GO Id | p-value | Name | Numbers of genes in this category | Numbers of genes in the signature. |
| GO:0000166 | (p=0.000001) | nucleotide binding | 943 | 74 |
| GO:0005515 | (p=0.000005) | protein binding | 2020 | 130 |
| GO:0004571 | (p=0.000053) | mannosyl-oligosaccharide 1,2-alpha-mannosidase activity | 6 | 4 |
| GO:0004579 | (p=0.000087) | dolichyl-diphosphooligosaccharide-protein glycotransferase activity | 3 | 3 |
| GO:0004888 | (p=0.000119) | transmembrane receptor activity | 76 | 12 |
| GO:0016740 | (p=0.000129) | transferase activity | 1124 | 76 |
| GO:0005201 | (p=0.000371) | extracellular matrix structural constituent | 41 | 8 |
| GO:0004812 | (p=0.000553) | tRNA ligase activity | 54 | 9 |
| GO:0003779 | (p=0.000738) | actin binding | 189 | 19 |
| GO:0008318 | (p=0.001420) | protein prenyltransferase activity | 12 | 4 |
| GO:0004528 | (p=0.001959) | phosphodiesterase I activity | 2 | 2 |
| GO:0004817 | (p=0.001959) | cysteine-tRNA ligase activity | 2 | 2 |
| GO:0016874 | (p=0.002683) | ligase activity | 180 | 17 |
| GO:0008565 | (p=0.003701) | protein transporter activity | 111 | 12 |
| GO:0016301 | (p=0.003889) | kinase activity | 692 | 46 |
| GO:0017124 | (p=0.004099) | SH3 domain binding | 8 | 3 |
| GO:0005096 | (p=0.004282) | GTPase activator activity | 113 | 12 |

Supplementary Table 1D. Genes present in nine GO Molecular Function categories that were shown to be functionally enriched (see Supplementary Table 1A, above)

| **Small GTPase mediated signal transduction** | | | | |  |  |
| --- | --- | --- | --- | --- | --- | --- |
| **GO:0007264** | |  |  |  |  |  |
| **(p=0.000043)** | |  |  |  |  |  |
|  |  |  |  |  |  |  |
| Affy Id | Unigene Id | Mean of intensities in PCTs | Mean of intensities in BCLs | Fold difference  PCT/BCL | Description | Gene symbol |
| 103231_at | Mm.358763 | 639.3 | 2713.3 | 0.236 | Ras homolog gene family, member H | Rhoh |
| 92534_at | Mm.247486 | 167.3 | 556.8 | 0.3 | GTP binding protein (gene overexpressed in skeletal muscle) | Gem |
| 104179_at | Mm.27308 | 376.3 | 1027.3 | 0.366 | ADP-ribosylation factor 6 | Arf6 |
| 92805_s_at | Mm.12723 | 138.9 | 360.3 | 0.386 | ADP-ribosylation factor-like 4 | Arl4 |
| 102283_at | Mm.124100 | 206.6 | 517.1 | 0.4 | T-cell lymphoma invasion and metastasis 1 | Tiam1 |
| 93347_at | Mm.220923 | 1795.9 | 3239.9 | 0.554 | RAB24, member RAS oncogene family | Rab24 |
| 97319_at | Mm.29467 | 591.1 | 1061.7 | 0.557 | Ras-related associated with diabetes | Rrad |
| 96238_at | Mm.1387 | 197.6 | 340.8 | 0.58 | RAB11a, member RAS oncogene family | Rab11a |
| 94998_at | Mm.27348 | 324.4 | 498.6 | 0.651 | V-ral simian leukemia viral oncogene homolog A (ras related) | Rala |
| 96262_at | Mm.29829 | 4962.3 | 7559.3 | 0.656 | RAB5C, member RAS oncogene family | Rab5c |
| 104112_at | Mm.21936 | 892.3 | 1306.9 | 0.683 | RAB21, member RAS oncogene family | Rab21 |
| 94105_at | Mm.1022 | 4450.3 | 6504.9 | 0.684 | Cell division cycle 42 homolog (S. cerevisiae) | Cdc42 |
| 94503_at | Mm.162811 | 5017.9 | 6645.8 | 0.755 | RAB8A, member RAS oncogene family | Rab8a |
| 97301_at | Mm.198264 | 4256.9 | 5397.5 | 0.789 | RIKEN cDNA D030017L14 gene | Rab14 |
| 99497_at | Mm.41637 | 2392.5 | 1486.2 | 1.61 | ADP-ribosylation factor interacting protein 2 | Arfip2 |
| 160731_at | Mm.26994 | 796.2 | 456.9 | 1.743 | RAB25, member RAS oncogene family | Rab25 |
| 101921_at | Mm.9221 | 746.2 | 396.5 | 1.882 | RAB4A, member RAS oncogene family | Rab4a |
| 101030_at | Mm.687 | 10119 | 4516.7 | 2.24 | Ras homolog gene family, member B | Rhob |

| **Intracellular signaling cascades** | | |  |  |  |  |
| --- | --- | --- | --- | --- | --- | --- |
| **GO:0007242** | |  |  |  |  |  |
| **(p=0.000001)** | |  |  |  |  |  |
|  |  |  |  |  |  |  |
| Affy Id | Unigene Id | Mean of intensities in PCTs | Mean of intensities in BCLs | Fold difference  PCT/BCL | Description | Gene symbol |
| 95893_at | Mm.3962 | 269.7 | 2404.7 | 0.112 | B lymphoid kinase | Blk |
| 100292_at | Mm.86361 | 175.6 | 965 | 0.182 | SH2 domain protein 2A | Sh2d2a |
| 102994_at | Mm.1550 | 178.4 | 636.3 | 0.28 | Signal transducer and activator of transcription 4 | Stat4 |
| 99876_at | Mm.7601 | 217.8 | 907.7 | 0.24 | Src-like adaptor | Sla |
| 102809_s_at | Mm.293753 | 594.8 | 2437.9 | 0.244 | Lymphocyte protein tyrosine kinase | Lck |
| 93662_s_at | Mm.8038 | 392.4 | 1568.4 | 0.25 | Zeta-chain (TCR) associated protein kinase | Zap70 |
| 102851_s_at | Mm.271799 | 755.6 | 2933.3 | 0.258 | Hemopoietic cell phosphatase | Hcph |
| 103596_at | Mm.291235 | 726.3 | 2484.1 | 0.292 | Diacylglycerol kinase, alpha | Dgka |
| 96331_at | Mm.252171 | 1629.9 | 5073.5 | 0.321 | Sorting nexin 2 | Snx2 |
| 103392_at | Mm.288206 | 1011.5 | 3152.2 | 0.321 | Adenylate cyclase 7 | Adcy7 |
| 98911_at | Mm.289657 | 1401.7 | 4199.8 | 0.334 | Janus kinase 1 | Jak1 |
| 98766_at | Mm.343694 | 179.3 | 505.2 | 0.355 | SH3-domain binding protein 5 (BTK-associated) | Sh3bp5 |
| 92998_at | Mm.179011 | 165.1 | 443.5 | 0.372 | Vav2 oncogene | Vav2 |
| 102283_at | Mm.124100 | 206.6 | 517.1 | 0.4 | T-cell lymphoma invasion and metastasis 1 | Tiam1 |
| 103662_at | Mm.2068 | 1425.5 | 3447.8 | 0.413 | Neutrophil cytosolic factor 4 | Ncf4 |
| 102028_at | Mm.248291 | 1667.5 | 3663.4 | 0.455 | Ras association (RalGDS/AF-6) domain family 5 | Rassf5 |
| 92668_at | Mm.4475 | 2007.8 | 4219.8 | 0.476 | Bruton agammaglobulinemia tyrosine kinase | Btk |
| 104679_at | Mm.3264 | 104.7 | 219.4 | 0.477 | TXK tyrosine kinase | Txk |
| 103349_at | Mm.317331 | 500.8 | 1035.8 | 0.483 | Yamaguchi sarcoma viral (v-yes-1) oncogene homolog | Lyn |
| 103279_at | Mm.235391 | 101.3 | 206.9 | 0.49 | SH2 domain protein 1A | Sh2d1a |
|  |  |  |  |  |  |  |
| 100511_at | Mm.243954 | 3656.6 | 7334.9 | 0.499 | RIKEN cDNA 6330406L22 gene | 6330406  L22Rik |
| 94556_at | Mm.294166 | 622 | 1373.9 | 0.453 | Sorting nexin 10 | Snx10 |
| 93647_at | Mm.324305 | 794.8 | 1774.4 | 0.448 | Gem-interacting protein | Gmip |
| 103028_at | Mm.339927 | 349.3 | 682.2 | 0.512 | IL2-inducible T-cell kinase | Itk |
| 97325_at | Mm.29515 | 964.1 | 1845.3 | 0.522 | PX domain containing serine/threonine kinase | Pxk |
| 102884_at | Mm.15105 | 2003.3 | 3802.3 | 0.527 | Inositol polyphosphate-5-phosphatase D | Inpp5d |
| 104431_at | Mm.329993 | 308.8 | 549.8 | 0.562 | Protein kinase C, theta | Prkcq |
| 92975_at | Mm.5012 | 422.4 | 737.4 | 0.573 | SH3-domain binding protein 2 | Sh3bp2 |
| 93939_at |  | 766.1 | 1325.4 | 0.578 | linker of T-cell receptor pathways | Lnk |
| 98001_at | Mm.3181 | 2084.6 | 3502.5 | 0.595 | Rho guanine nucleotide exchange factor (GEF) 1 | Arhgef1 |
| 100425_at | Mm.122843 | 6129.1 | 9329.3 | 0.657 | Spleen tyrosine kinase | Syk |
| 99467_at | Mm.259653 | 1329.5 | 1971.4 | 0.674 | RAS p21 protein activator 1 | Rasa1 |
| 100423_f_at |  | 1242.3 | 1774.5 | 0.7 | signal transducer and activator of transcription 5A | Stat5a |
| 103247_at | Mm.20449 | 679.5 | 309.3 | 2.197 | Membrane protein, palmitoylated 3 (MAGUK p55 subfamily member 3) | Mpp3 |

| **tRNA aminoacylation (protein translation)** | | | |  |  |  |
| --- | --- | --- | --- | --- | --- | --- |
| **GO:0006418** | |  |  |  |  |  |
| **(p=0.000049)** | |  |  |  |  |  |
|  |  |  |  |  |  |  |
| Affy Id | Unigene Id | Mean of intensities in PCTs | Mean of intensities in BCLs | Fold difference  PCT/BCL | Description | Gene symbol |
|  |  |  |  |  |  |  |
| 94941_at | Mm.217616 | 221.7 | 399.8 | 0.555 | Eukaryotic translation initiation factor 2 alpha kinase 4 | Eif2ak4 |
| 104702_at | Mm.2437 | 918.2 | 642.8 | 1.428 | BING4 protein | Bing4 |
| 93752_at | Mm.21118 | 7521.8 | 5137.9 | 1.464 | Isoleucine-tRNA synthetase | Iars |
| 93564_at | Mm.145488 | 4589.5 | 2985 | 1.538 | Tyrosyl-tRNA synthetase | Yars |
| 104048_at | Mm.125659 | 1926.7 | 1248.1 | 1.544 | Cysteinyl-tRNA synthetase | Cars |
| 96628_at | Mm.154511 | 4506.2 | 2853.5 | 1.579 | Glutamyl-prolyl-tRNA synthetase | Eprs |
| 98936_at | Mm.28688 | 2688.6 | 1672.6 | 1.607 | Seryl-aminoacyl-tRNA synthetase 1 | Sars1 |
| 93270_at | Mm.250004 | 7737.1 | 4534.9 | 1.706 | Storage granule protein 23 | Gars |
| 103630_at | Mm.312170 | 7094.6 | 3964.3 | 1.79 | Leucyl-tRNA synthetase | Lars |

| **Cell surface receptor linked signal transduction** | | | | |  |  |
| --- | --- | --- | --- | --- | --- | --- |
| **GO:0007166** | |  |  |  |  |  |
| **(p=0.000010)** | |  |  |  |  |  |
|  |  |  |  |  |  |  |
| Affy Id | Unigene Id | Mean of intensities in PCTs | Mean of intensities in BCLs | Fold difference  PCT/BCL | Description | Gene symbol |
| 102778_at | Mm.1355 | 493.5 | 9141.6 | 0.054 | CD79A antigen (immunoglobulin-associated alpha) | Cd79a |
| 99030_at | Mm.389 | 266.8 | 2005.6 | 0.133 | Interleukin 7 receptor | Il7r |
| 102939_s_at | Mm.260994 | 182.4 | 1312.7 | 0.139 | CD22 antigen | Cd22 |
| 96525_at | Mm.26658 | 139.4 | 592.4 | 0.235 | Interleukin 10 receptor, alpha | Il10ra |
| 102971_at |  | 160.9 | 607.5 | 0.265 | CD3 antigen, epsilon polypeptide | Cd3e |
| 100001_at |  | 153.7 | 541.8 | 0.284 | CD3 antigen, gamma polypeptide | Cd3g |
| 92683_at | Mm.4527 | 132.6 | 462.3 | 0.287 | CD3 antigen, delta polypeptide | Cd3d |
| 103866_at | Mm.229108 | 1212.9 | 3173.9 | 0.382 | Phosphatidylinositol 3-kinase catalytic delta polypeptide | Pik3cd |
| 161689_f_at | Mm.1349 | 308.6 | 724 | 0.426 | Interleukin 1 receptor, type II | Il1r2 |
| 92955_at | Mm.4944 | 231.9 | 526.4 | 0.441 | Interleukin 3 receptor, alpha chain | Il3ra |
| 102287_at | Mm.217308 | 115.1 | 234.8 | 0.49 | CD3 antigen, zeta polypeptide | Cd3z |
| 100425_at | Mm.122843 | 6129.1 | 9329.3 | 0.657 | Spleen tyrosine kinase | Syk |
| 93681_at | Mm.36416 | 458.1 | 195.4 | 2.344 | Frizzled homolog 2 (Drosophila) | Fzd2 |

| **Apoptosis** |  |  |  |  |  |  |
| --- | --- | --- | --- | --- | --- | --- |
| **GO:0006915** | |  |  |  |  |  |
| **(p=0.001079)** | |  |  |  |  |  |
|  |  |  |  |  |  |  |
| Affy Id | Unigene Id | Mean of intensities in PCTs | Mean of intensities in BCLs | Fold difference  PCT/BCL | Description | Gene symbol |
| 103255_at | Mm.347811 | 235.6 | 1515.6 | 0.155 | Tnf receptor-associated factor 5 | Traf5 |
| 104093_at | Mm.234003 | 1291 | 5704.6 | 0.226 | Lymphocyte specific 1 | Lsp1 |
| 94186_at | Mm.239514 | 137.5 | 433.4 | 0.317 | Tnf receptor-associated factor 1 | Traf1 |
| 99392_at | Mm.116683 | 112.4 | 326.2 | 0.345 | Tumor necrosis factor, alpha-induced protein 3 | Tnfaip3 |
| 93869_s_at |  | 1317.6 | 3818.5 | 0.345 | B-cell leukemia/lymphoma 2 related protein A1d | Bcl2a1d |
| 98436_s_at | Mm.34405 | 468.6 | 1275.4 | 0.367 | Caspase 3, apoptosis related cysteine protease | Casp3 |
| 102914_s_at | Mm.196770 | 2631.8 | 6933.9 | 0.38 | B-cell leukemia/lymphoma 2 related protein A1d | Bcl2a1a |
| 97890_at | Mm.28405 | 1009.7 | 2608 | 0.387 | Serum/glucocorticoid regulated kinase | Sgk |
| AFFX-MurFAS_at | Mm.1626 | 171.2 | 402 | 0.426 | Tumor necrosis factor receptor superfamily, member 6 | Tnfrsf6 |
| 92962_at | Mm.271833 | 202.6 | 441.2 | 0.459 | Tumor necrosis factor receptor superfamily, member 5 | Tnfrsf5 |
| 98868_at |  | 110 | 233.7 | 0.471 | B-cell leukemia/lymphoma 2 | Bcl2 |
| 98427_s_at | Mm.256765 | 3607.5 | 7391.6 | 0.488 | Ubiquitin-conjugating enzyme E2D 3 (UBC4/5 homolog, yeast) | Nfkb1 |
| 98499_s_at | Mm.35687 | 340.7 | 677 | 0.503 | Caspase 7 | Casp7 |
| 94458_at | Mm.281379 | 169 | 306.4 | 0.552 | Caspase 6 | Casp6 |
| 160696_at | Mm.274425 | 693.7 | 1172 | 0.592 | Cytotoxic granule-associated RNA binding protein 1 | Tia1 |
| 95040_at | Mm.29816 | 266.3 | 434 | 0.614 | Programmed cell death 6 interacting protein | Pdcd6ip |
| 103796_at | Mm.220289 | 814.8 | 1316.2 | 0.619 | Apoptotic protease activating factor 1 | Apaf1 |
| 93064_at | Mm.159777 | 1610.2 | 2352.2 | 0.685 | BCL2/adenovirus E1B 19kDa-interacting protein 1, NIP2 | Bnip2 |
| 96008_at |  | 19863.7 | 13193.7 | 1.506 | defender against cell death 1 | Dad1 |
| 93564_at | Mm.145488 | 4589.5 | 2985 | 1.538 | Tyrosyl-tRNA synthetase | Yars |
| 95486_at | Mm.24997 | 6479.2 | 4084.3 | 1.586 | Phosphatidylserine receptor | Ptdsr |
| 101030_at | Mm.687 | 10119 | 4516.7 | 2.24 | Ras homolog gene family, member B | Rhob |
| 93439_f_at | Mm.336104 | 882.4 | 328.7 | 2.685 | PRKC, apoptosis, WT1, regulator | Pawr |
| **B cell receptor signaling pathway** | | | |  | | |
| **GO:0050853** |  |  |  |  |  | |
| **(p=0.000878)** |  |  |  |  |  |  |
|  |  |  |  |  |  |  |
| Affy Id | Unigene Id | Mean intensities in PCTs | Mean intensities in BCLs | Fold difference  PCT/BCL | Description | Gene symbol |
| 102778_at | Mm.1355 | 493.5 | 9141.6 | 0.054 | CD79A antigen (immunoglobulin-associated alpha) | Cd79a |
| 93584_at | Mm.351746 | 865.8 | 7957.8 | 0.109 | Immunoglobulin heavy chain 6 (heavy chain of IgM) | Igh-6 |
| 100425_at | Mm.122843 | 6129.1 | 9329.3 | 0.657 | Spleen tyrosine kinase | Syk |

| **antigen processing, exogenous antigen via MHC class II** | | | | |  |  |
| --- | --- | --- | --- | --- | --- | --- |
| **GO:0019886** | |  |  |  |  |  |
| **(p=0.000554)** | |  |  |  |  |  |
|  |  |  |  |  |  |  |
| Affy Id | Unigene Id | Mean intwnsities in PCTs | Mean intensities in BCLs | Fold difference  PCT/BCL | Description | Gene symbol |
| 104429_at |  | 200.9 | 2098.5 | 0.096 | histocompatibility 2, O region beta locus | H2-Ob |
| 102274_at | Mm.116 | 287 | 1314.7 | 0.218 | Histocompatibility 2, O region alpha locus | H2-Oa |
| 162346_f_at | Mm.16373 | 311.6 | 964.3 | 0.323 | Histocompatibility 2, class II, locus DMa | H2-DMa |
| 97444_at | Mm.30241 | 5536.5 | 10609.5 | 0.522 | Interferon gamma inducible protein 30 | Ifi30 |
| 93092_at | Mm.16373 | 2996.8 | 6255.4 | 0.479 | Histocompatibility 2, class II, locus DMa | H2-DMa |

| **lymph node development** | | |  |  |  |  |
| --- | --- | --- | --- | --- | --- | --- |
| **GO:0048535** | |  |  |  |  |  |
| **(p=0.001690)** | |  |  |  |  |  |
|  |  |  |  |  |  |  |
| Affy Id | Unigene Id | Mean intensities in PCTs | Mean intensities in BCLs | Fold difference  PCT/BCL | Description | Gene symbol |
| 102940_at | Mm.1715 | 380.2 | 3423.8 | 0.111 | Lymphotoxin B | Ltb |
| 99030_at | Mm.389 | 266.8 | 2005.6 | 0.133 | Interleukin 7 receptor | Il7r |
| 102630_s_at | Mm.87787 | 120.8 | 382.6 | 0.316 | Lymphotoxin A | Lta |
| 93416_at | Mm.249221 | 196.1 | 484.3 | 0.405 | Tumor necrosis factor (ligand) superfamily, member 11 | Tnfsf11 |
| 98427_s_at | Mm.256765 | 3607.5 | 7391.6 | 0.488 | Ubiquitin-conjugating enzyme E2D 3 (UBC4/5 homolog, yeast) | Nfkb1 |

| **positive regulation of B cell differentiation** | | | |  |  |  |
| --- | --- | --- | --- | --- | --- | --- |
| **GO:0045579** | |  |  |  |  |  |
| **(p=0.000878)** | |  |  |  |  |  |
|  |  |  |  |  |  |  |
| Affy Id | Unigene Id | Mean intensities in PCTs | Mean intensities in BCLs | Fold difference  PCT/BCL | Description | Gene symbol |
| 102884_at | Mm.15105 | 2003.3 | 3802.3 | 0.527 | Inositol polyphosphate-5-phosphatase D | Inpp5d |
| 100425_at | Mm.122843 | 6129.1 | 9329.3 | 0.657 | Spleen tyrosine kinase | Syk |
| 100423_f_at |  | 1242.3 | 1774.5 | 0.7 | signal transducer and activator of transcription 5A | Stat5a |
