# Supplementary material for: Gene expression profiling reveals different pathways related to Abl and other genes that cooperate with c-Myc in a model of plasma cell neoplasia
Source: BMC Genomics. 2007 Aug 31;8:302. doi: 10.1186/1471-2164-8-302 (PMC2040348; doi:10.1186/1471-2164-8-302)
Supplement: Additional file 2 — Supplementary Tables 2. Genes that showed significant (p < 0.001) differences in expression between rapid-forming plasma cell tumors (ABPC and ABLMYCPC) and slow-forming plasma cell tumors (TEPC, IL6PC and KiPC). Supplementary Table 2A. Genes that showed more than 2-fold higher expression in rapid-forming plasma cell tumors than in slow-forming plasma cell tumors. Supplementary Table 2B. Genes that showed more than 2-fold higher expression in slow-forming plasma cell tumors than in rapid-forming plasma cell tumors. Two tables showing lists of genes that showed significant (p < 0.001) differences in expression between rapid- and slow-forming plasma cell tumors. [file 1471-2164-8-302-S2.doc]

**Additional file 2**

Supplementary Tables 2. Genes that showed significant (p<0.001) differences in expression between rapid-forming plasma cell tumors (ABPC and ABLMYCPC) and slow-forming plasma cell tumors (TEPC, IL6PC and KiPC)

Supplementary Table 2A. Genes that showed more than 2-fold higher expression in rapid-forming plasma cell tumors than in slow-forming plasma cell tumors

| Affy Id | Unigene Id | Rapid | Slow | Fold difference  Rapid/Slow | Description | Gene symbol | p-value |
| --- | --- | --- | --- | --- | --- | --- | --- |
| 93391_at | Mm.6253 | 5641.1 | 403.1 | 13.994 | Crystallin, gamma S | Crygs | < 1e-07 |
| 92203_s_at | Mm.290897 | 3568.1 | 386 | 9.244 | CD6 antigen | Cd6 | 2.00E-07 |
| 96591_at | Mm.3057 | 4373.4 | 474.2 | 9.223 | Reelin | Reln | 5.50E-06 |
| 99475_at | Mm.4132 | 9118.7 | 1057.2 | 8.625 | Suppressor of cytokine signaling 2 | Socs2 | < 1e-07 |
| 94432_at | Mm.149029 | 6565.2 | 980.4 | 6.696 | Beta galactoside alpha 2,6 sialyltransferase 1 | St6gal1 | < 1e-07 |
| 101972_at | Mm.9495 | 10068.7 | 1517.4 | 6.635 | Napsin A aspartic peptidase | Napsa | < 1e-07 |
| 92204_at | Mm.290897 | 2102.6 | 370.8 | 5.67 | CD6 antigen | Cd6 | 2.00E-07 |
| 160420_r_at | Mm.287784 | 984.7 | 186.1 | 5.291 | Tubulin, alpha 3 | Tuba3 | < 1e-07 |
| 92832_at | Mm.130 | 6941.3 | 1361.4 | 5.099 | Suppressor of cytokine signaling 1 | Socs1 | < 1e-07 |
| 100880_at | Mm.254851 | 13750.1 | 2848.3 | 4.827 | Diabetic nephropathy-like protein mRNA, partial sequence | Dnr12 | < 1e-07 |
| 98822_at | Mm.358664 | 4954.9 | 1080.5 | 4.586 | Interferon, alpha-inducible protein | G1p2 | < 1e-07 |
| 161511_f_at | Mm.358664 | 812.7 | 186.8 | 4.351 | Interferon, alpha-inducible protein | G1p2 | < 1e-07 |
| 98433_at | Mm.235081 | 2661.9 | 718.2 | 3.706 | BH3 interacting domain death agonist | Bid | < 1e-07 |
| 104750_at | Mm.24769 | 3770.1 | 1018.5 | 3.702 | Olfactory receptor 56 | Olfr56 | < 1e-07 |
| 102203_at | Mm.1001 | 1566 | 426.5 | 3.672 | Placental protein 11 related | Pp11r | 6.40E-06 |
| 102906_at | Mm.15793 | 5476.9 | 1546.5 | 3.541 | T-cell specific GTPase | Tgtp | 9.00E-07 |
| 103432_at |  | 4266.8 | 1225.2 | 3.483 | interferon-stimulated protein (20 kDa) | Isg20 | 0.00011 |
| 100706_f_at | Mm.27840 | 900 | 269.1 | 3.344 | Scm-like with four mbt domains 2 | Sfmbt2 | 0.000209 |
| 96172_at | Mm.333048 | 9174 | 2747.2 | 3.339 | Immune associated nucleotide 1 | Ian1 | < 1e-07 |
| 100133_at | Mm.370182 | 4085.5 | 1225.5 | 3.334 | Fyn proto-oncogene | Fyn | < 1e-07 |
| 100467_at | Mm.4925 | 6231.6 | 1885.8 | 3.304 | Lymphoblastomic leukemia | Lyl1 | < 1e-07 |
| 97204_s_at | Mm.248046 | 1648.5 | 503.4 | 3.275 | DnaJ (Hsp40) homolog, subfamily D, member 1 | Dnajd1 | 7.00E-07 |
| 160829_at | Mm.3117 | 7231.5 | 2242.3 | 3.225 | Pleckstrin homology-like domain, family A, member 1 | Phlda1 | 0.000425 |
| 95974_at | Mm.250 | 2277.2 | 724.7 | 3.142 | Guanylate nucleotide binding protein 1 | Gbp1 | 0.000215 |
| 103049_at |  | 3174.8 | 1065.2 | 2.98 | Unknown |  | 2.57E-05 |
| 97969_at | Mm.3095 | 1116.9 | 376.6 | 2.966 | Nuclear receptor subfamily 1, group H, member 4 | Nr1h4 | 5.00E-07 |
| 94431_at | Mm.149029 | 1215.2 | 414 | 2.935 | Beta galactoside alpha 2,6 sialyltransferase 1 | St6gal1 | 3.00E-07 |
| 92581_at | Mm.10530 | 7492.6 | 2554.5 | 2.933 | Acetyl-Coenzyme A dehydrogenase, medium chain | Acadm | < 1e-07 |
| 161126_at | Mm.343880 | 2312.8 | 803.3 | 2.879 | Gene model 288, (NCBI) |  | 0.000861 |
| 162329_r_at |  | 1803.4 | 627.5 | 2.874 | secretory leukocyte protease inhibitor | Slpi | 0.000838 |
| 102851_s_at | Mm.271799 | 1310.4 | 470.1 | 2.787 | Hemopoietic cell phosphatase | Hcph | 1.08E-05 |
| 95518_at | Mm.25311 | 3290.5 | 1199.6 | 2.743 | RIKEN cDNA 1810015C04 gene | 1810015  C04Rik | 1.86E-05 |
| 93561_at | Mm.20313 | 361.7 | 137.1 | 2.638 | RIKEN cDNA 0710001P09 gene | Chchd6 | 3.00E-07 |
| 93924_f_at | Mm.270295 | 6145.3 | 2364.8 | 2.599 | Tubulin, alpha 7 | Tuba7 | < 1e-07 |
| 101900_at | Mm.269426 | 482.9 | 186.4 | 2.591 | Cyclin-dependent kinase inhibitor 2B (p15, inhibits CDK4) | Cdkn2b | 0.000386 |
| 160695_i_at | Mm.228 | 2517 | 998.4 | 2.521 | Homer homolog 2 (Drosophila) | Homer2 | 8.16E-05 |
| 103367_at | Mm.1853 | 889.1 | 358.7 | 2.479 | UDP-N-acetyl-alpha-D-galactosamine:(N-acetylneuraminyl)-galactosylglucosylceramide-beta-1, 4-N-acetylgalactosaminyltransferase | Galgt1 | 0.000899 |
| 100468_g_at | Mm.4925 | 15588.9 | 6372.8 | 2.446 | Lymphoblastomic leukemia | Lyl1 | 6.00E-07 |
| 101875_at | Mm.295009 | 933.6 | 386 | 2.419 | Protein phosphatase 2, regulatory subunit B (B56), delta isoform | Ppp2r5d | 0.000321 |
| 104063_at | Mm.151940 | 915 | 378.8 | 2.416 | COX11 homolog, cytochrome c oxidase assembly protein (yeast) | Cox11 | 0.000319 |
| 103202_at | Mm.371069 | 11108.4 | 4623.5 | 2.403 | Transcribed locus |  | 1.16E-05 |
| 103963_f_at |  | 1737 | 726.1 | 2.392 | interferon-inducible GTPase | Iigp-  pending | 0.000477 |
| 101048_at | Mm.130953 | 4367.3 | 1830.9 | 2.385 | Protein tyrosine phosphatase, receptor type, C | Ptprc | 0.00015 |
| 101498_at | Mm.183042 | 3195.6 | 1346.2 | 2.374 | Inositol (myo)-1(or 4)-monophosphatase 1 | Impa1 | < 1e-07 |
| 94392_f_at |  | 556.6 | 235.1 | 2.368 | angiogenin | Ang | 5.10E-06 |
| 160607_at | Mm.299254 | 323.7 | 136.8 | 2.366 | Par-3 (partitioning defective 3) homolog (C. elegans) | Pard3 | 1.55E-05 |
| 160712_r_at | Mm.24506 | 2106.2 | 894.7 | 2.354 | RIKEN cDNA E130012A19 gene | E130012  A19Rik | 2.00E-07 |
| 100013_at | Mm.45558 | 3520.3 | 1507.4 | 2.335 | Interferon-induced protein 35 | Ifi35 | < 1e-07 |
| 98624_at | Mm.3865 | 5326.4 | 2289.8 | 2.326 | RNA-binding region (RNP1, RRM) containing 1 | Rnpc1 | 5.00E-06 |
| 93962_at | Mm.333868 | 2455 | 1064.3 | 2.307 | RAS-related protein-1a | Rap1a | < 1e-07 |
| 92644_s_at | Mm.52109 | 1636.6 | 715.6 | 2.287 | Myeloblastosis oncogene | Myb | 0.000206 |
| 104276_at | Mm.22213 | 759.5 | 332.7 | 2.283 | GLI pathogenesis-related 2 | Glipr2 | 0.000223 |
| 160948_at | Mm.1567 | 1450.6 | 637.2 | 2.277 | Protein phosphatase 3, catalytic subunit, gamma isoform | Ppp3cc | < 1e-07 |
| 94774_at | Mm.218770 | 1516 | 666.5 | 2.275 | Interferon activated gene 202B | Ifi202b | 0.000994 |
| 104592_i_at | Mm.24001 | 1298.4 | 571.1 | 2.274 | Myocyte enhancer factor 2C | Mef2c | 4.74E-05 |
| 102877_at | Mm.14874 | 832.4 | 368.3 | 2.26 | Granzyme B | Gzmb | 0.000305 |
| 94939_at | Mm.316861 | 9821.9 | 4360.2 | 2.253 | CD53 antigen | Cd53 | 3.40E-06 |
| 101716_at |  | 257.8 | 114.6 | 2.25 | Unknown |  | 0.000171 |
| 98136_at | Mm.18652 | 1326.1 | 590.8 | 2.245 | Spermine synthase | Sms | 1.20E-05 |
| 92398_at | Mm.4079 | 320.3 | 143.6 | 2.231 | CDNA sequence BC026744 | BC026744 | 2.73E-05 |
| 103035_at | Mm.207996 | 7479.7 | 3365.9 | 2.222 | Transporter 1, ATP-binding cassette, sub-family B (MDR/TAP) | Tap1 | < 1e-07 |
| 101918_at | Mm.248380 | 1754.9 | 791.5 | 2.217 | Transforming growth factor, beta 1 | Tgfb1 | 0.00033 |
| 97409_at | Mm.29938 | 1494.8 | 678.8 | 2.202 | Interferon inducible protein 1 | Ifi1 | 5.00E-07 |
| 102880_at | Mm.176725 | 358.4 | 166.5 | 2.153 | RIKEN cDNA E130103I17 gene | E130103  I17Rik | 0.000239 |
| 103881_at | Mm.210305 | 1166.7 | 544.4 | 2.143 | RIKEN cDNA 1110013G13 gene | 1110013  G13Rik | < 1e-07 |
| 97445_at |  | 3749.5 | 1767.5 | 2.121 | peptidylprolyl isomerase D (cyclophilin D) | Ppid | 1.00E-07 |
| 101459_at | Mm.8137 | 3274.2 | 1544.3 | 2.12 | Chromodomain helicase DNA binding protein 1 | Chd1 | < 1e-07 |
| 103039_at | Mm.16234 | 597 | 283.2 | 2.108 | Integrin alpha 5 (fibronectin receptor alpha) | Itga5 | 0.000144 |
| 97208_at | Mm.27706 | 1119.1 | 533.5 | 2.098 | Ash2 (absent, small, or homeotic)-like (Drosophila) | Ash2l | 0.000308 |
| 99000_at | Mm.38172 | 763.2 | 366.9 | 2.08 | Mitogen activated protein kinase 7 | Mapk7 | 6.15E-05 |
| 100538_at | Mm.276325 | 7347.8 | 3535.9 | 2.078 | Superoxide dismutase 1, soluble | Sod1 | < 1e-07 |
| 97772_at | Mm.4183 | 1868.3 | 902.8 | 2.069 | Plasminogen activator, urokinase | Plau | 8.29E-05 |
| 92489_at | Mm.252599 | 3381.8 | 1635.4 | 2.068 | Immunity-associated protein | Imap38 | < 1e-07 |
| 104177_at | Mm.24045 | 358.9 | 175 | 2.051 | RIKEN cDNA 2510004L01 gene | 2510004  L01Rik | 0.000511 |
| 101486_at | Mm.787 | 10726.7 | 5269.5 | 2.036 | Proteasome (prosome, macropain) subunit, beta type 10 | Psmb10 | < 1e-07 |
| 103259_at | Mm.2078 | 365.9 | 179.9 | 2.034 | Growth factor independent 1 | Gfi1 | 1.30E-05 |
| 103258_at | Mm.2074 | 631.6 | 311.1 | 2.03 | Lymphocyte antigen 75 | Ly75 | 2.07E-05 |
| 100023_at | Mm.4594 | 2855.2 | 1408.4 | 2.027 | Myeloblastosis oncogene-like 2 | Mybl2 | 9.80E-06 |
| 98030_at | Mm.295578 | 487.9 | 242 | 2.016 | Tripartite motif protein 30 | Trim30 | 0.000168 |

Supplementary Table 2B. Genes that showed more than 2 fold higher expression in slow-forming plasma cell tumors than in rapid-forming plasma cell tumors

| Affy Id | Unigene Id | Rapid | Slow | Fold difference  Rapid/Slow | Description | Gene symbol | Parametric p-value |
| --- | --- | --- | --- | --- | --- | --- | --- |
| AFFX-MURINE_B2_at |  | 1216.9 | 11598.4 | 0.105 | Unknown |  | < 1e-07 |
| 99238_at |  | 259.9 | 1686.6 | 0.154 | Unknown |  | < 1e-07 |
| 97282_at | Mm.270157 | 2155.4 | 12439.4 | 0.173 | Melanoma antigen | Mela | 4.11E-05 |
| 94154_at | Mm.12800 | 151.6 | 839.4 | 0.181 | Thyroglobulin | Tgn | < 1e-07 |
| 104428_s_at | Mm.2918 | 583.6 | 2799.1 | 0.208 | Megakaryocyte-associated tyrosine kinase | Matk | < 1e-07 |
| 160553_at | Mm.878 | 1819.9 | 7645 | 0.238 | Lymphocyte antigen 6 complex, locus D | Ly6d | 1.17E-05 |
| 96597_at | Mm.3086 | 282 | 1177 | 0.24 | Plasmacytoma expressed transcript 2 | Pet2 | 0.000278 |
| 104198_at | Mm.27519 | 331.5 | 1273.3 | 0.26 | RIKEN cDNA E030027H19 gene | E030027  H19Rik | 5.30E-06 |
| 92214_at | Mm.113590 | 137.4 | 526.9 | 0.261 | Cathepsin W | Ctsw | 9.86E-05 |
| 96712_at | Mm.273295 | 609.5 | 2153.3 | 0.283 | SPARC related modular calcium binding 1 | Smoc1 | 7.00E-07 |
| 96198_at | Mm.28561 | 270.1 | 912.6 | 0.296 | Protein kinase C, zeta | Prkcz | < 1e-07 |
| 92665_f_at | Mm.335676 | 1408.7 | 4706 | 0.299 | X-linked lymphocyte-regulated complex | Xlr | 0.000131 |
| 93705_at | Mm.86425 | 283.8 | 934.6 | 0.304 | Cholinergic receptor, nicotinic, beta polypeptide 1 (muscle) | Chrnb1 | 1.00E-07 |
| 94803_at | Mm.43358 | 152.3 | 495.2 | 0.308 | Pre B-cell leukemia transcription factor 1 | Pbx1 | < 1e-07 |
| 102833_at | Mm.14547 | 168.8 | 546.7 | 0.309 | Chromobox homolog 2 (Drosophila Pc class) | Cbx2 | 5.00E-07 |
| 96724_r_at | Mm.200783 | 339.7 | 1082.3 | 0.314 | Synovial sarcoma, X breakpoint 2 interacting protein | Ssx2ip | 2.00E-07 |
| 96599_at | Mm.284370 | 136 | 432.2 | 0.315 | RIKEN cDNA C330016K18 gene | C330016  K18Rik | < 1e-07 |
| 102682_at | Mm.1390 | 395.4 | 1199.6 | 0.33 | Eph receptor A8 | Epha8 | < 1e-07 |
| 162332_f_at |  | 128.6 | 385 | 0.334 | expressed sequence AI790651 | AI790651 | 4.00E-07 |
| 161050_at | Mm.292000 | 494.9 | 1477.8 | 0.335 | 10 days neonate cerebellum cDNA, RIKEN full-length enriched library, clone:6530415D11 product:unknown EST, full insert sequence |  | 0.000132 |
| 100286_at | Mm.8369 | 245.5 | 721.6 | 0.34 | Macrophage stimulating 1 (hepatocyte growth factor-like) | Mst1 | 0.00012 |
| 101516_at | Mm.247265 | 1171.8 | 3380.1 | 0.347 | CD59a antigen | Cd59a | 1.00E-07 |
| 92205_at |  | 327 | 940.9 | 0.348 | ESTs, Highly similar to insulin receptor substrate-2 [M.musculus] |  | 2.31E-05 |
| 104045_at | Mm.21485 | 174.6 | 490.1 | 0.356 | DNA segment, Chr 10, Brigham & Women's Genetics 0791 expressed | D10Bwg  0791e | 8.00E-07 |
| 102801_at |  | 231.3 | 646 | 0.358 | expressed sequence AI461847 | AI461847 | 0.000165 |
| 92521_at | Mm.196564 | 294 | 803.5 | 0.366 | AT motif binding factor 1 | Atbf1 | < 1e-07 |
| 93913_at | Mm.258708 | 188 | 504.3 | 0.373 | Early B-cell factor 3 | Ebf3 | 5.90E-06 |
| 104214_at |  | 346.6 | 927.6 | 0.374 | solute carrier family 7 (cationic amino acid transporter, y+ system), member 8 | Slc7a8 | < 1e-07 |
| 95030_at | Mm.10516 | 204.1 | 535.4 | 0.381 | Prolactin receptor | Prlr | 5.40E-06 |
| 96963_s_at |  | 561.1 | 1451 | 0.387 | immunoglobulin kappa chain variable 28 (V28) | Igk-V28 | 0.000149 |
| 99059_at | Mm.3963 | 247.9 | 639.4 | 0.388 | E74-like factor 3 | Elf3 | 6.80E-06 |
| 93575_at | Mm.20461 | 327 | 842.5 | 0.388 | Gamma-glutamyl hydrolase | Ggh | 4.97E-05 |
| 93861_f_at |  | 5021.2 | 12888.5 | 0.39 | Mus musculus, clone IMAGE:5053066, mRNA, partial cds |  | 1.30E-06 |
| 99532_at |  | 3036.9 | 7597.3 | 0.4 | transducer of ErbB-2.1 | Tob1 | 1.80E-06 |
| 102221_at | Mm.230301 | 2052.3 | 5116 | 0.401 | Synaptogyrin 1 | Syngr1 | 1.00E-07 |
| 99366_at | Mm.5675 | 4406.8 | 10955.9 | 0.402 | RIKEN cDNA E030024M05 gene | E030024  M05Rik | < 1e-07 |
| 100566_at | Mm.309617 | 270.1 | 671.2 | 0.402 | Insulin-like growth factor binding protein 5 | Igfbp5 | 3.47E-05 |
| 100295_at | Mm.38214 | 1438.2 | 3568.2 | 0.403 | Ig rearranged lambda-chain (NC19-F12) mRNA Vl2-Jl1 region | 2010309  G21Rik | 4.80E-06 |
| 101030_at | Mm.687 | 6059.8 | 14988.6 | 0.404 | Ras homolog gene family, member B | Rhob | < 1e-07 |
| 96926_at | Mm.30162 | 298.4 | 738.7 | 0.404 | SPARC related modular calcium binding 2 | Smoc2 | 1.20E-06 |
| 102922_at | Mm.340968 | 349.7 | 854.7 | 0.409 | Phosphatidylinositol transfer protein, cytoplasmic 1 | Pitpnc1 | < 1e-07 |
| 104255_at | Mm.329322 | 100.9 | 243.6 | 0.414 | Formin-family protein FHOS2 | FHOS2 | 8.28E-05 |
| 96605_at | Mm.27061 | 5101.5 | 12296.8 | 0.415 | RIKEN cDNA 0610011I04 gene | 0610011  I04Rik | 3.50E-05 |
| 102623_at | Mm.12903 | 246 | 590.7 | 0.416 | Sema domain, immunoglobulin domain (Ig), short basic domain, secreted, (semaphorin) 3 F | Sema3f | 1.90E-05 |
| 161666_f_at | Mm.1360 | 2655.3 | 6300.5 | 0.421 | Growth arrest and DNA-damage-inducible 45 beta | Gadd45b | 1.00E-07 |
| 93353_at | Mm.18888 | 697.1 | 1656.1 | 0.421 | Lumican | Lum | 0.000822 |
| 93940_at | Mm.9122 | 2385.9 | 5600.1 | 0.426 | Paraoxonase 3 | Pon3 | 0.000603 |
| 104725_at | Mm.826 | 1303.6 | 3049.8 | 0.427 | Ras homolog gene family, member Q | Rhoq | 1.00E-07 |
| 162256_r_at | Mm.361469 | 776.5 | 1786.6 | 0.435 | Transcribed locus |  | 2.00E-07 |
| 96180_at | Mm.20954 | 164 | 375.7 | 0.437 | Regulator of G-protein signaling 5 | Rgs5 | 0.000115 |
| 103460_at | Mm.21697 | 2548.5 | 5739.8 | 0.444 | DNA-damage-inducible transcript 4 | Ddit4 | 1.93E-05 |
| 99067_at | Mm.3982 | 727.4 | 1631.9 | 0.446 | Growth arrest specific 6 | Gas6 | 0.000881 |
| 94804_at | Mm.43358 | 844.6 | 1883 | 0.449 | Pre B-cell leukemia transcription factor 1 | Pbx1 | < 1e-07 |
| 93903_at | Mm.370168 | 334.3 | 743 | 0.45 | Activin receptor IIB | Acvr2b | 0.000106 |
| 103299_at | Mm.203915 | 2384.6 | 5274.6 | 0.452 | Expressed sequence AI132321 | AI132321 | 1.38E-05 |
| 99582_at | Mm.4259 | 694.2 | 1536.7 | 0.452 | Tumor-associated calcium signal transducer 1 | Tacstd1 | 0.000165 |
| 161465_r_at |  | 1876.3 | 4135.3 | 0.454 | zinc finger protein 94 | Zfp94 | 4.10E-06 |
| 103006_at | Mm.1566 | 1042 | 2288 | 0.455 | Activating transcription factor 5 | Atf5 | 0.000215 |
| 160678_at | Mm.21950 | 118.2 | 260 | 0.455 | Transmembrane 4 superfamily member 12 | Tm4sf12 | 0.000704 |
| 94958_at | Mm.274708 | 155.2 | 340.7 | 0.456 | RIKEN cDNA 1110013L07 gene | 1110013  L07Rik | 9.00E-06 |
| 104134_at | Mm.279228 | 2064.9 | 4523.7 | 0.456 | Ganglioside-induced differentiation-associated-protein 2 | Gdap2 | 2.07E-05 |
| 100576_at | Mm.597 | 4459.3 | 9703.3 | 0.46 | Platelet-activating factor acetylhydrolase, isoform 1b, alpha1 subunit | Pafah1b3 | 1.00E-07 |
| 93404_g_at | Mm.6306 | 830.9 | 1807 | 0.46 | ATPase, Ca++ transporting, ubiquitous | Atp2a3 | 8.00E-07 |
| 99381_at | Mm.56933 | 134.1 | 291.6 | 0.46 | Fucosyltransferase 1 | Fut1 | 0.000675 |
| 102637_at | Mm.200775 | 124 | 267.7 | 0.463 | Transforming growth factor, beta receptor III | Tgfbr3 | 1.20E-06 |
| 103029_at | Mm.1605 | 749.7 | 1609.4 | 0.466 | Programmed cell death 4 | Pdcd4 | < 1e-07 |
| 97138_at | Mm.237935 | 232.5 | 495.3 | 0.469 | CDNA sequence BC026657 | BC026657 | < 1e-07 |
| 100336_s_at |  | 398.7 | 840.5 | 0.474 | bone gamma-carboxyglutamate protein 2 | Bglap2 | 2.33E-05 |
| 98859_at | Mm.46354 | 900.2 | 1893.1 | 0.476 | Acid phosphatase 5, tartrate resistant | Acp5 | 0.000808 |
| 98435_at | Mm.3440 | 1522.8 | 3187.8 | 0.478 | Adenylosuccinate synthetase like 1 | Adssl1 | 0.000119 |
| 93860_i_at |  | 4398.8 | 9211.6 | 0.478 | Mus musculus, clone IMAGE:5053066, mRNA, partial cds |  | 0.000442 |
| 93206_g_at | Mm.388 | 1280 | 2664.4 | 0.48 | Adenosine deaminase | Ada | 0.000547 |
| 96603_at | Mm.27035 | 223.4 | 464.8 | 0.481 | Quiescin Q6 | Qscn6 | 1.00E-07 |
| 98462_s_at | Mm.279599 | 129.9 | 269.3 | 0.482 | RIKEN cDNA 1200014P03 gene | 1200014  P03Rik | 1.00E-07 |
| 104129_at |  | 261.4 | 539.5 | 0.485 | cytochrome P450 CYP4F13 | Cypf13 | < 1e-07 |
| 96494_at | Mm.274579 | 149.1 | 306.8 | 0.486 | RIKEN cDNA 4930429H24 gene | 4930429  H24Rik | 1.58E-05 |
| 97853_at | Mm.105331 | 2566.3 | 5244.9 | 0.489 | Small nuclear RNA activating complex, polypeptide 3 | Psip1 | < 1e-07 |
| 102779_at | Mm.1360 | 1751 | 3562.6 | 0.491 | Growth arrest and DNA-damage-inducible 45 beta | Gadd45b | < 1e-07 |
| 103647_at | Mm.290516 | 2157.6 | 4384.8 | 0.492 | RIKEN cDNA C130097A14 gene | Glb1 | 4.00E-07 |
| 96723_f_at | Mm.200783 | 2035.9 | 4136.8 | 0.492 | Synovial sarcoma, X breakpoint 2 interacting protein | Ssx2ip | 1.00E-06 |
| 161754_f_at |  | 477.3 | 970.5 | 0.492 | galactosidase, beta 1 | Glb1 | 2.10E-06 |
| 92305_s_at | Mm.208700 | 765.9 | 1554.6 | 0.493 | POU domain, class 2, transcription factor 2 | Pou2f2 | 2.30E-06 |
| 97918_at | Mm.243632 | 2125.9 | 4273.7 | 0.497 | Expressed sequence AA536743 | AA536743 | 2.00E-07 |
