# Supplementary material for: Gene expression profiling reveals different pathways related to Abl and other genes that cooperate with c-Myc in a model of plasma cell neoplasia
Source: BMC Genomics. 2007 Aug 31;8:302. doi: 10.1186/1471-2164-8-302 (PMC2040348; doi:10.1186/1471-2164-8-302)
Supplement: Additional file 3 — Supplementary Figure 1. Quantitative RT-PCR of relative mRNA content in 13 mouse B-cell lymphomas and plasma cell tumors for 9 key genes. Results of quantitative RT-PCR validation of relative mRNA content in 13 mouse B-cell lymphomas and plasma cell tumors for 9 key genes: c-Myc, Socs1, Socs2, Abl1, Jak1, Xbp1, Sdc1, Irf4, Pax5 (BSAP) and the "housekeeping" gene, GAPDH. [file 1471-2164-8-302-S3.pdf]

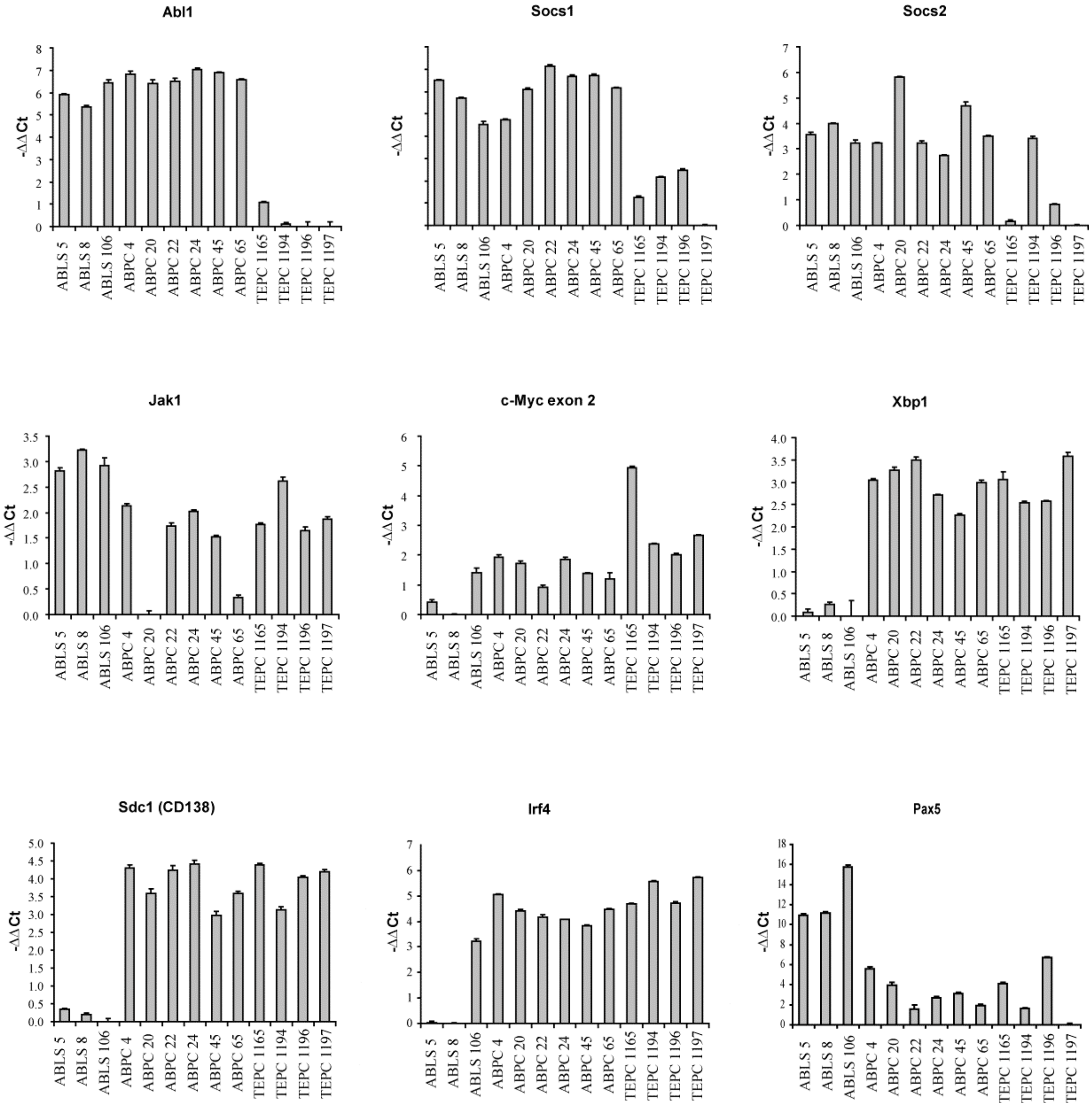

Additional file 3, Supplementary Figure 1. RNAs from the 13 indicated tumors were assayed for relative mRNA content for 9 key genes: c-*Myc, Socs1, Socs2, Abl1, Jak1, Xbp1, Sdc1, Irf4,* and *Pax5 (BSAP)* as well as for the “housekeeping gene,” GAPDH, using primers designed in consultation with the manufacturer (Applied Biosystems, Foster City, CA). All data were normalized to GAPDH, and these values were then calibrated against the sample with the lowest expression and represented as –ΔΔct values with standard errors.
