# Supplementary material for: Gene expression profiling reveals different pathways related to Abl and other genes that cooperate with c-Myc in a model of plasma cell neoplasia
Source: BMC Genomics. 2007 Aug 31;8:302. doi: 10.1186/1471-2164-8-302 (PMC2040348; doi:10.1186/1471-2164-8-302)
Supplement: Additional file 4 — Supplementary Tables 3. Genes with significant (p < 0.001) differences in gene expression associated with site of chromosomal translocation in two sample t-tests. Supplementary Table 3A. Genes that showed significant differences in expression between ABPCs with T(12;15) and ABPCs with T(6;15). Supplementary Table 3B. Genes that showed significant differences in expression between TEPCs with T(12;15 class I) and TEPC T(12;15 class II) chromosome translocations. Two tables showing lists of genes that showed significant (p < 0.001) differences in expression between similar subclasses of plasma cell tumors that differ in site of Myc-activating chromosomal translocation. [file 1471-2164-8-302-S4.doc]

**Additional file 4.**

Supplementary Tables 3. Genes with significant (p<0.001) differences in gene expression associated with site of chromosomal translocation in two sample t-tests

Supplementary Table 3A. Genes that showed significant differences in expression between ABPCs with T(12;15) and ABPCs with T(6;15)

| Affy Id | T(12;15) | T(6;15) | Fold difference | Description | Gene symbol | Unigene Id | p-value |
| --- | --- | --- | --- | --- | --- | --- | --- |
| 96704_at | 9797.3 | 1521.9 | 6.438 | Stratifin | Sfn | Mm.44482 | 0.000922 |
| 104337_f_at | 2921.1 | 459.2 | 6.361 | Plakophilin 2 | Pkp2 | Mm.2252 | 0.000901 |
| 104338_r_at | 1065.1 | 167.8 | 6.347 | Plakophilin 2 | Pkp2 | Mm.2252 | 2.50E-05 |
| 103340_at | 368.9 | 118.2 | 3.121 | Rhesus blood group CE and D | Rhced | Mm.195461 | 1.63E-05 |
| 102942_at | 292.3 | 108.9 | 2.684 | RIKEN cDNA B230396K10 gene | 2900056 B14Rik | Mm.37803 | 0.000492 |
| 96787_at | 265.1 | 120.3 | 2.204 | Serine (or cysteine) proteinase inhibitor, clade A (alpha-1 antiproteinase, antitrypsin), member 10 | Serpina10 | Mm.29094 | 0.000581 |
| 93623_at | 219.6 | 100 | 2.196 | dystrophin, muscular dystrophy | Dmd |  | 0.000165 |
| 101901_at | 1714.2 | 798 | 2.148 | Mannose-P-dolichol utilization defect 1 | Mpdu1 | Mm.89579 | 0.000229 |
| 103842_at | 228.9 | 110.6 | 2.07 | DEAD (Asp-Glu-Ala-Asp) box polypeptide 3, Y-linked | Ddx3y | Mm.302938 | 0.000477 |
| 99864_at | 174 | 100 | 1.74 | Adenosine A2b receptor | Adora2b | Mm.40740 | 0.000994 |
| 97549_at | 259.5 | 584.1 | 0.444 | Cofilin 2, muscle | Cfl2 | Mm.276826 | 0.000941 |
| 95063_at | 2606.4 | 6752.9 | 0.386 | Cell division cycle associated 7 | Cdca7 | Mm.270676 | 0.000478 |
| 102676_at | 119.8 | 360.5 | 0.332 | Von Hippel-Lindau syndrome homolog | Vhlh | Mm.29407 | 2.23E-05 |
| 92315_at | 591.6 | 1836 | 0.322 | Schlafen 4 | Slfn4 | Mm.38192 | 0.000561 |

Supplementary Table 3B. Genes that showed significant differences in expression between TEPCs with T(12;15 class I) and TEPC T(12;15 class II) chromosome translocations

| Affy Id | T(12;15 class I) | T(12;15 class II) | Fold difference | Description | Gene symbol | Unigene Id | p-value |
| --- | --- | --- | --- | --- | --- | --- | --- |
| 95425_at | 2500.1 | 935 | 2.674 | Acetyl-Coenzyme A dehydrogenase, long-chain | Acadl | Mm.2445 | 0.00035 |
| 103006_at | 2948.9 | 1106.4 | 2.665 | Activating transcription factor 5 | Atf5 | Mm.1566 | 0.000651 |
| 101772_r_at | 2233.7 | 849.9 | 2.628 | Protocadherin alpha 11 | Pcdha4 | Mm.308500 | 0.000608 |
| 98470_at | 325.7 | 128.9 | 2.527 | Solute carrier family 25 (mitochondrial carrier, brain), member 14 | Slc25a14 | Mm.34953 | 0.000403 |
| 104144_at | 4176.3 | 1695.6 | 2.463 | GTP binding protein 2 | Gtpbp2 | Mm.22147 | 8.34E-05 |
| 92818_at | 1693.4 | 834.6 | 2.029 | Ubiquitin-activating enzyme E1C | Ube1c | Mm.277626 | 0.000759 |
| 104651_at | 1129.4 | 582.5 | 1.939 | Sorting nexin 14 | Snx14 | Mm.163017 | 0.000269 |
| 92648_at | 1230.9 | 647.3 | 1.902 | Syntaxin binding protein3 | Stxbp3 | Mm.316894 | 0.000682 |
| 93274_at | 7818.4 | 4423.2 | 1.768 | CDC-like kinase 1 | Clk1 | Mm.1761 | 0.000354 |
| 93337_at | 3323 | 1943.4 | 1.71 | Vacuolar protein sorting 4b (yeast) | Vps4b | Mm.18705 | 0.000182 |
| 94478_at | 3775.1 | 2267.1 | 1.665 | Zinc finger protein 367 | Zfp367 | Mm.300065 | 0.000576 |
| 99143_at | 4887.9 | 2946.5 | 1.659 | Transcribed locus |  | Mm.370529 | 0.000291 |
| 94508_at | 2661.6 | 1657.5 | 1.606 | RIKEN cDNA 1810020E01 gene | 1810020  E01Rik | Mm.28963 | 0.000363 |
| 103875_at | 2257.2 | 1496.8 | 1.508 | Expressed sequence AW552001 | AW552001 | Mm.21175 | 0.000386 |
| 104022_at | 1569 | 1050.9 | 1.493 | M-phase phosphoprotein 10 (U3 small nucleolar ribonucleoprotein) | Mphosph  10 | Mm.26973 | 0.000527 |
| 103402_at | 618.7 | 444.9 | 1.391 | Transmembrane 7 superfamily member 3 | Tm7sf3 | Mm.278037 | 0.000582 |
| 96636_at | 3692.9 | 2914.5 | 1.267 | Dynactin 5 | Dctn5 | Mm.27270 | 0.000835 |
| 101431_at | 120.7 | 151.6 | 0.796 | Retinol dehydrogenase 5 | Rdh5 | Mm.358629 | 0.000501 |
| 94260_at | 6695 | 9886.8 | 0.677 | RIKEN cDNA 2510010K19 gene | 3110040  D16Rik | Mm.248843 | 0.000339 |
| 161064_f_at | 600 | 903.6 | 0.664 | PHD finger protein 7 | Phf7 | Mm.5348 | 0.000471 |
| 94380_at | 804.9 | 1219 | 0.66 | Insulin degrading enzyme | Ide | Mm.28366 | 0.000373 |
| 103768_at | 612 | 935.8 | 0.654 | DNA segment, Chr 19, ERATO Doi 678, expressed | D19Ert  d678e | Mm.294908 | 0.000992 |
| 161408_r_at | 166.5 | 260.9 | 0.638 | Dishevelled 2, dsh homolog (Drosophila) | Dvl2 | Mm.5114 | 0.000673 |
| 103975_at | 102.1 | 164.5 | 0.621 | Gremlin 2 homolog, cysteine knot superfamily (Xenopus laevis) | Grem2 | Mm.25760 | 0.000408 |
| 101510_at | 6391.8 | 10595.8 | 0.603 | Proteasome (prosome, macropain) 28 subunit, alpha | Psme1 | Mm.830 | 0.000565 |
| 96310_at | 395.4 | 634 | 0.624 | Myelin basic protein | Mbp | Mm.252063 | 0.000136 |
| 93300_at | 132.4 | 217.9 | 0.608 | Transforming growth factor, beta 2 | Tgfb2 | Mm.18213 | 0.000166 |
| 96729_at | 101.4 | 202.4 | 0.501 | Eukaryotic translation initiation factor 4E binding protein 2 | Eif4ebp2 | Mm.259516 | 0.000315 |
| 95614_at | 644.1 | 1540.4 | 0.418 | Chromobox homolog 5 (Drosophila HP1a) | Cbx5 | Mm.262059 | 0.000717 |
