# Supplementary material for: Gene expression profiling reveals different pathways related to Abl and other genes that cooperate with c-Myc in a model of plasma cell neoplasia
Source: BMC Genomics. 2007 Aug 31;8:302. doi: 10.1186/1471-2164-8-302 (PMC2040348; doi:10.1186/1471-2164-8-302)
Supplement: Additional file 5 — Supplementary Figure 2. Meta-analysis of global gene expression of 67 mouse samples (including 5 different subgroups of plasma cell tumors and B-cell lymphomas) combined with 123 human samples (including 5 different subgroups of multiple myelomas and tonsilar B cells). Heat map derived from a meta-analysis of a combination of expression data from human and mouse plasma cell dyscrasias. [file 1471-2164-8-302-S5.pdf]

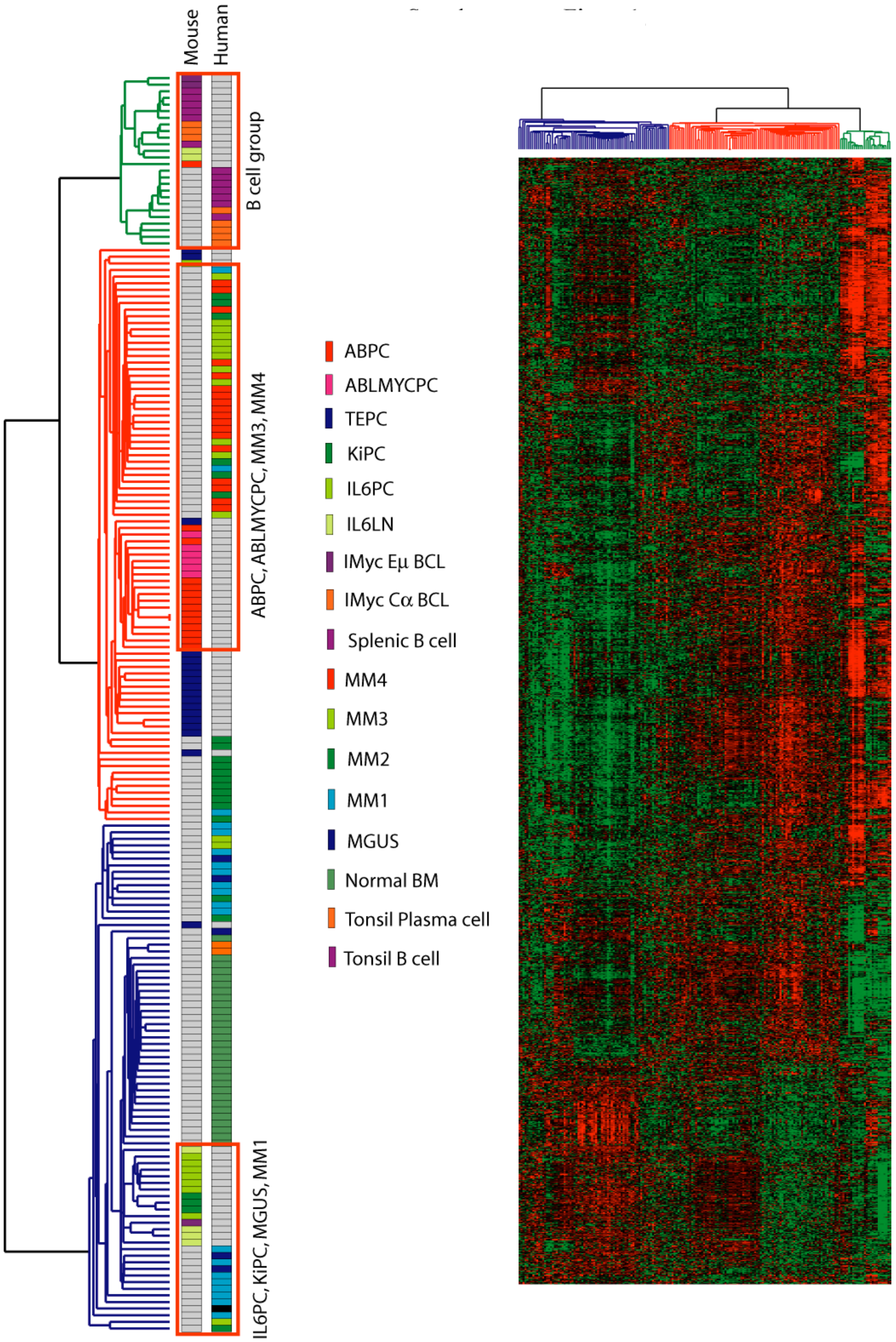

Additional file 5, Supplementary Figure 2. A total of 190 samples comprised of 123 human samples and 67 mouse samples were used for this meta-analysis. Human samples are composed of normal bone marrow samples, MGUS, 4 different stages of multiple myeloma (MM1, MM2, MM3 and MM4), tonsil plasma and tonsil B cells. Mouse samples are composed of 5 different plasmacytomas groups (ABPC, ABLMYC, TEPC, KiPC and IL6PC), 3 B cell lymphoma groups (BLL, BL and IL6 LN) and iMycEμ pre-malignant splenic B cells. The human and mouse data were normalized and standardized separately. The genes for cluster analysis were selected by one-way ANOVA analysis of combined orthologues, and 1488 genes that showed significant differences ($p < 1 \times 10^{-7}$) were used for cluster analysis.
